# Metabolic pathways linking air pollution to osteoarthritis: Insights from a prospective cohort

**DOI:** 10.1371/journal.pone.0341125

**Published:** 2026-01-20

**Authors:** Bojun Zhang, Ping Li, Chaojun Yang, Zhixing Fan

**Affiliations:** 1 Department of Anesthesia, Tianjin Hospital, Tianjin, China; 2 Department of Cardiology, the First College of Clinical Medical Sciences, China Three Gorges University, Yichang, China; 3 Institute of Cardiovascular Diseases, China Three Gorges University, Yichang, China; 4 Hu Bei Clinical Research Center for Ischemic Cardiovascular Disease, Yichang, China; Mazandaran University of Medical Sciences, IRAN, ISLAMIC REPUBLIC OF

## Abstract

**Objective:**

To investigate metabolic pathways linking air pollution exposure to osteoarthritis (OA) development and quantify their mediating role in disease pathogenesis.

**Methods:**

This prospective cohort study utilized UK Biobank data from 220,872 participants. Air pollution exposure was assessed using land use regression models for PM₂.₅, PM₁₀, PM₂.₅ ₋ ₁₀, NO₂, and NOₓ, with composite scores constructed. Nuclear magnetic resonance metabolomics profiling quantified 251 circulating metabolites. Elastic net regression identified air pollution-related metabolic signatures. Cox proportional hazards models assessed associations of air pollution related metabolic profiles with incident OA. Causal mediation analysis quantified metabolic pathway mediation using counterfactual methods.

**Results:**

During follow-up, 40,399 participants (18.3%) developed incident OA. Elastic net regression identified 50 metabolites associated with air pollution scores, encompassing lipoprotein subclasses (26%), fatty acids (16%), amino acids (12%), and inflammatory biomarkers. Air pollution-related metabolic signatures showed stronger associations with OA risk (HR 1.095, 95% CI: 1.082–1.108 per IQR increase) than air pollution scores alone (HR 1.030, 95% CI: 1.018–1.042). Effects were most pronounced for knee OA (HR 1.140, 95% CI: 1.118–1.162). Causal mediation analysis revealed that metabolic signatures mediated 21.04% (95% CI: 16.52%−41.95%) of the air pollution-OA association.

**Conclusion:**

Metabolic pathways significantly mediate air pollution-OA associations, providing novel insights into environmental contributions to musculoskeletal health and identifying potential therapeutic targets for prevention strategies.

## 1. Introduction

Osteoarthritis (OA) represents one of the most prevalent degenerative joint diseases worldwide, imposing an enormous burden on global public health systems and significantly affecting individuals’ quality of life. Recent systematic analyses have revealed alarming trends in the global OA burden, with the number of affected individuals surging from approximately 527.8 million in 1990–607 million by 2021, reflecting a substantial increase of nearly 15% over three decades [1, 2]. The age-standardized prevalence rate has demonstrated a consistent upward trajectory, rising from 6,393.1 per 100,000 population in 1990–6,967.3 per 100,000 in 2021, with corresponding increases in disability-adjusted life years (DALYs) from 222.8 to 244.5 per 100,000 population [3, 4]. Notably, knee OA accounts for the largest proportion of the disease burden, with global prevalence reaching 374.7 million cases and generating 12.01 million DALYs in 2021 [5, 6]. The disease exhibits pronounced gender disparities, with women consistently experiencing higher prevalence, incidence, and disability rates across all age groups, and projections indicate that the global burden will continue to escalate, potentially reaching 765 million cases by 2060 [7, 8]. These epidemiological trends underscore the urgent need to identify novel risk factors and elucidate previously unrecognized pathogenic mechanisms to develop more effective prevention strategies for this debilitating condition.

Beyond traditional risk factors such as aging, obesity, and mechanical stress, emerging epidemiological evidence has identified air pollution as a novel environmental determinant of OA development. Recent large-scale cohort studies have demonstrated compelling associations between long-term air pollutant exposure and increased OA risk [9]. A nationwide case-crossover study in China found that short-term exposure to air pollutants significantly increased OA-related hospital admissions [10]. Furthermore, air pollution exposure not only increases OA incidence but also adversely affects survival outcomes in established disease [11]. The biological mechanisms underlying these associations may be elucidated through metabolomic approaches, as mounting evidence suggests that air pollution induces profound alterations in circulating metabolic profiles that could mediate disease pathogenesis. High-resolution metabolomics studies have revealed that air pollutants trigger systematic perturbations in oxidative stress responses, inflammatory cascades, and lipid metabolism [12, 13]. Studies utilizing elastic net regression and nuclear magnetic resonance (NMR) spectroscopy have successfully identified air pollution-related metabolic signatures comprising hundreds of metabolites, demonstrating significant associations with chronic diseases including respiratory, cardiovascular, and neurological conditions [14–17]. Concurrently, metabolomic investigations in OA have revealed substantial dysregulation in lipid metabolism, amino acid metabolism, and energy homeostasis pathways fundamental to cartilage maintenance and joint inflammation [18–20]. However, the specific metabolic pathways linking air pollution exposure to OA pathogenesis remain largely unexplored, representing a critical knowledge gap that limits our understanding of environmental contributions to joint disease and targeted preventive intervention development.

We conducted a comprehensive prospective cohort study utilizing UK Biobank data to systematically investigate the metabolic pathways linking air pollution exposure to OA development. Our primary objectives were threefold: first, to identify air pollution-related metabolic signatures using advanced NMR metabolomics profiling and elastic network regression; second, to evaluate prospective associations between these metabolic signatures and incident OA risk; and third, to quantify the mediating role of metabolic pathways in the air pollution-OA relationship through mediation analyses.

## 2. Methods

### 2.1. Study design

This prospective cohort study was conducted within the UK Biobank, a large-scale population-based biomedical repository established to investigate genetic and environmental determinants of disease in middle-aged and older adults. The UK Biobank recruited over 500,000 participants aged 40–69 years from 22 assessment centers across England, Wales, and Scotland between 2006 and 2010. Participants underwent comprehensive baseline assessments including detailed questionnaires, physical measurements, and biological sample collection for metabolomic profiling. The study protocol, data collection procedures, and cohort characteristics have been extensively documented in previous publications. All participants provided written informed consent, and the study received ethical approval from the North West Multi-Centre Research Ethics Committee (REC ID: 21/NW/0157). The present analysis was conducted under UK Biobank application number 170605. Data were accessed in April 2024, and individual identities cannot be identified through data.

**Fig 1:**
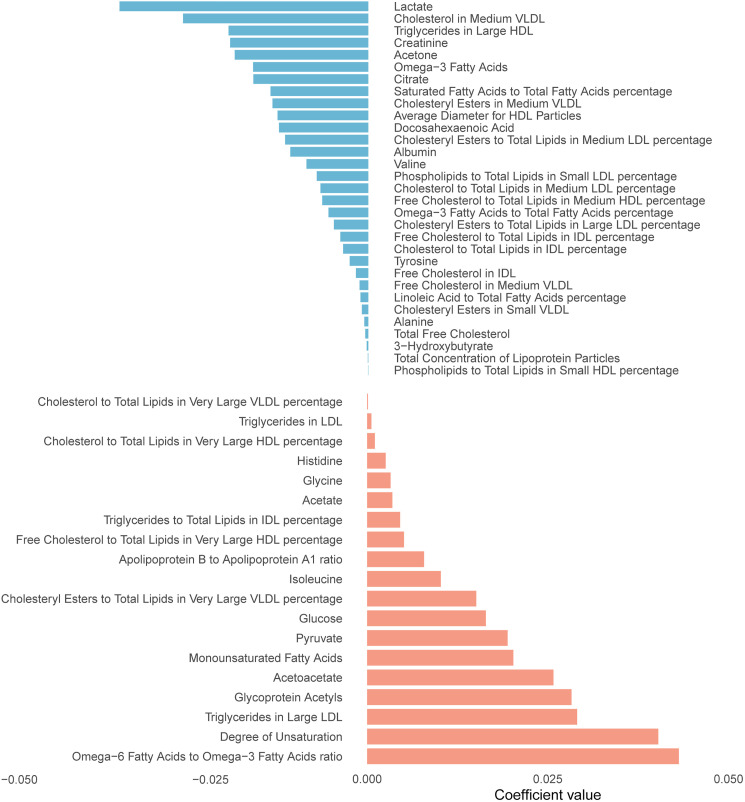
Metabolites ranked from the highest to the lowest elastic network positive and negative regression coefficients for air pollution.

From the initial cohort of 502,132 participants, we identified 275,233 individuals with available NMR metabolomics data. We excluded participants with missing metabolomics data (n = 26,052), incomplete air pollution exposure information (n = 17,272), or prevalent OA at baseline (n = 10,041). After applying these selection criteria, 220,872 participants comprised the final analytical cohort for prospective analysis of incident OA (Fig 1).

### 2.2. Air pollution

We evaluated five major air pollutants: fine particulate matter with aerodynamic diameter ≤2.5 μm (PM₂.₅), coarse particulate matter with aerodynamic diameter ≤10 μm (PM₁₀), particulate matter with aerodynamic diameter between 2.5–10 μm (PM₂.₅ ₋ ₁₀), nitrogen dioxide (NO₂), and nitrogen oxides (NOₓ). Air pollution concentrations were derived from the Small Area Health Statistics Unit at Imperial College London, which employed land use regression models incorporating satellite-derived estimates, ground-level monitoring data, and geographic covariates including traffic density, population density, and land cover characteristics.

To capture the combined effect of multiple pollutant exposures, we constructed an air pollution score using the following formula [11]: air pollution score = (βPM₂.₅ × PM₂.₅ + βPM₁₀ × PM₁₀ + βPM₂.₅ ₋ ₁₀ × PM₂.₅ ₋ ₁₀ + βNO₂ × NO₂ + βNOₓ × NOₓ) × (5/sum of the β coefficients), where β coefficients represent the relative weights of each pollutant. This composite score provides an integrated measure of overall air pollution burden, accounting for the potentially synergistic effects of multiple pollutants to which participants were simultaneously exposed.

### 2.3. NMR metabolic biomarker

Plasma metabolomic profiling was conducted using high-throughput NMR spectroscopy on baseline blood samples collected from UK Biobank participants. The NMR metabolomics platform, developed by Nightingale Health Ltd (Helsinki, Finland), employed a standardized, automated analysis pipeline to quantify 251 circulating metabolites. This comprehensive metabolomic panel encompasses lipoprotein subclass concentrations and particle compositions, low-molecular-weight metabolites including amino acids, ketone bodies, and glycolysis-related compounds, as well as fatty acid profiles and inflammatory biomarkers. The detailed methodological protocols for NMR spectroscopy are publicly accessible through the UK Biobank data showcase.

### 2.4. Osteoarthritis

The primary outcome was incident OA, defined as the first recorded diagnosis during follow-up among participants without prevalent disease at baseline. Cases were identified through linkage to national health registries. OA diagnoses were ascertained using International Classification of Diseases 10th and 9th Revision (ICD-10/ICD-9) diagnostic codes, encompassing overall OA and site-specific subtypes including knee, hip, and hand OA as detailed in sTable 1. Follow-up time was calculated from baseline assessment to first OA diagnosis, death, loss to follow-up, or study end (December 31, 2022), whichever occurred first.

**Table 1:** Baseline characteristics of participants stratified by incident osteoarthritis status.

Characteristic	Level	Overall (*n* = 220872)	Control (*n* = 180473)	Osteoarthritis (*n* = 40399)	*P*
Age (years)		56.45 ± 8.09	55.74 ± 8.13	59.58 ± 7.09	<0.001
Sex (%)	Female	116751(52.86)	93329(51.71)	23422(57.98)	<0.001
	Male	104121(47.14)	87144(48.29)	16977(42.02)	
Race (%)	Other	11054(5.00)	9522(5.28)	1532(3.79)	<0.001
	White	209818(95.00)	170951(94.72)	38867(96.21)	
Education (%)	No university degree	150773(68.26)	119801(66.38)	30972(76.67)	<0.001
	University degree	70099(31.74)	60672(33.62)	9427(23.33)	
Income (%)	<£18,000	52084(23.58)	39516(21.90)	12568(31.11)	<0.001
	>£18,000	168788(76.42)	140957(78.10)	27831(68.89)	
BMI (kg/m^2^)		27.46 ± 4.73	27.15 ± 4.56	28.89 ± 5.20	<0.001
	<25 kg/m^2^	71458(32.35)	62272(34.50)	9186(22.74)	<0.001
	25 ~ 29 kg/m^2^	95655(43.31)	78520(43.51)	17135(42.41)	
	≥30 kg/m^2^	53759(24.34)	39681(21.99)	14078(34.85)	
Physical activity (%)	Low	41920(18.98)	34038(18.86)	7882(19.51)	<0.001
	Moderate	88615(40.12)	73126(40.52)	15489(38.34)	
	High	90337(40.90)	73309(40.62)	17028(42.15)	
Smoke (%)	Never	121327(54.93)	101133(56.04)	20194(49.99)	<0.001
	Previous	76507(34.64)	60440(33.49)	16067(39.77)	
	Current	23038(10.43)	18900(10.47)	4138(10.24)	
Alcohol (%)	Never	9692(4.39)	7684(4.26)	2008(4.97)	<0.001
	Previous	7722(3.50)	5923(3.28)	1799(4.45)	
	Current	203458(92.12)	166866(92.46)	36592(90.58)	
DASH diet	No	92094(41.70)	75207(41.67)	16887(41.80)	0.64
	Yes	128778(58.30)	105266(58.33)	23512(58.20)	
History of diabetes mellitus (%)	No	209101(94.67)	171709(95.14)	37392(92.56)	<0.001
	Yes	11771(5.33)	8764(4.86)	3007(7.44)	
History of hypertension (%)	No	153286(69.40)	129250(71.62)	24036(59.50)	<0.001
	Yes	67586(30.60)	51223(28.38)	16363(40.50)	
History of CVD (%)	No	203745(92.25)	167999(93.09)	35746(88.48)	<0.001
	Yes	17127(7.75)	12474(6.91)	4653(11.52)	
History of cancer (%)	No	201069(91.03)	165056(91.46)	36013(89.14)	<0.001
	Yes	19803(8.97)	15417(8.54)	4386(10.86)	
PM_2.5_ (μg/m^3^)		9.99 ± 1.06	9.99 ± 1.06	10.01 ± 1.06	<0.001
PM_10_ (μg/m^3^)		16.18 ± 1.88	16.18 ± 1.89	16.17 ± 1.86	0.524
PM_2.5–10_ (μg/m^3^)		6.41 ± 0.89	6.41 ± 0.89	6.41 ± 0.89	0.621
NO_2_ (μg/m^3^)		26.47 ± 7.50	26.48 ± 7.54	26.44 ± 7.31	0.348
NO_x_ (μg/m^3^)		43.87 ± 15.54	43.84 ± 15.58	44.02 ± 15.35	0.029

BMI: Body mass index; DASH: Dietary approaches to stop hypertension; CVD: Cardiovascular disease.

### 2.5. Covariate

Potential confounding variables were identified based on previous literature and included demographic characteristics, lifestyle factors, comorbidities, and socioeconomic indicators collected at baseline assessment. Demographic variables included age (continuous), sex (male/female), and self-reported ethnicity (White/Other). Socioeconomic status was assessed through educational attainment (university degree/no university degree) and annual household income (<£18,000/ ≥ £18,000). Anthropometric measurements included body mass index (BMI), calculated from height and weight measured by trained personnel using standardized protocols. Lifestyle factors encompassed physical activity levels categorized as low, moderate, or high based on the International Physical Activity Questionnaire, smoking status (never/previous/current), alcohol consumption frequency (never/previous/current), and adherence to Dietary Approaches to Stop Hypertension (DASH) diet patterns. Medical history variables included self-reported physician-diagnosed diabetes mellitus, hypertension, cardiovascular disease (CVD), and cancer, supplemented by prescription medication data and hospital records where available. Missing covariate data were handled using multiple imputation by chained equations.

### 2.6. Statistical analyses

Baseline characteristics were summarized using descriptive statistics, with continuous variables presented as means ± standard deviations or medians with IQRs. Categorical variables were expressed as frequencies and percentages. Between-group comparisons were performed using Student’s t-test or Mann-Whitney U test for continuous variables and chi-square tests for categorical variables.

To derive air pollution-related metabolic signatures, we implemented elastic net regularized regression, a machine learning approach combining Ridge and LASSO penalties to handle high-dimensional metabolomic data while addressing multicollinearity. Metabolites with non-zero regression coefficients were retained, and pollutant-specific metabolic signature scores were calculated as weighted linear combinations: Metabolic Signature Score = Σ(βᵢ × metaboliteᵢ). Spearman correlation analyses examined associations between derived metabolic signatures and individual air pollutant concentrations. Air pollution and metabolite data were standardized using z-score transformation. Air pollution and metabolic signature scores were also categorized into quartiles (Q1-Q4), using the lowest quartile (Q1) as the reference category for risk estimation.

Cox proportional hazards models assessed associations between exposures and incident OA risk across three sequential adjustment models: Model 1 (demographic and socioeconomic factors), Model 2 (additional lifestyle factors), and Model 3 (further including medical history). HRs with 95% confidence intervals were calculated per interquartile range (IQR) increase and across quartiles. Kaplan-Meier (KM) survival curves were constructed to visualize cumulative incidence of OA across exposure quartiles, with log-rank tests used to assess between-group differences in survival distributions. Dose-response relationships were examined using restricted cubic splines (RCS).

Causal mediation analysis employed the “CMAverse” package to quantify the proportion of air pollution-OA associations mediated by metabolic signatures, using counterfactual-based methods with bootstrap resampling (n = 200). Subgroup analyses stratified by covariates, with interaction testing via likelihood ratio tests. Sensitivity analyses comprised: (1) complete case analysis; (2) exclusion of participants developing OA within two years; and (3) exclusion of those with baseline chronic diseases (diabetes, hypertension, CVD, and cancer).

All analyses used R software version 4.4.3, with statistical significance at P < 0.05 and Bonferroni correction for multiple comparisons where appropriate.

## 3. Results

### 3.1. Baseline characteristics

Among the 220,872 participants included in the final analysis, 40,399 (18.3%) developed incident OA during follow-up (Table 1). Participants who developed OA were significantly older (59.58 ± 7.09 vs 55.74 ± 8.13 years), more likely to be female (57.98% vs 51.71%), and had higher body mass index (BMI) (28.89 ± 5.20 vs 27.15 ± 4.56 kg/m²) with greater obesity prevalence (34.85% vs 21.99%, all P < 0.001). Regarding air pollution exposure, the OA group had slightly higher PM₂.₅ (10.01 ± 1.06 vs 9.99 ± 1.06 μg/m³, P < 0.001) and NOₓ concentrations (44.02 ± 15.35 vs 43.84 ± 15.58 μg/m³, P = 0.029). The OA group also exhibited lower educational attainment, reduced income levels, and higher prevalence of baseline comorbidities including diabetes, hypertension, CVD, and cancer.

### 3.2. Identification of metabolic signatures for air pollution

**Fig 2:**
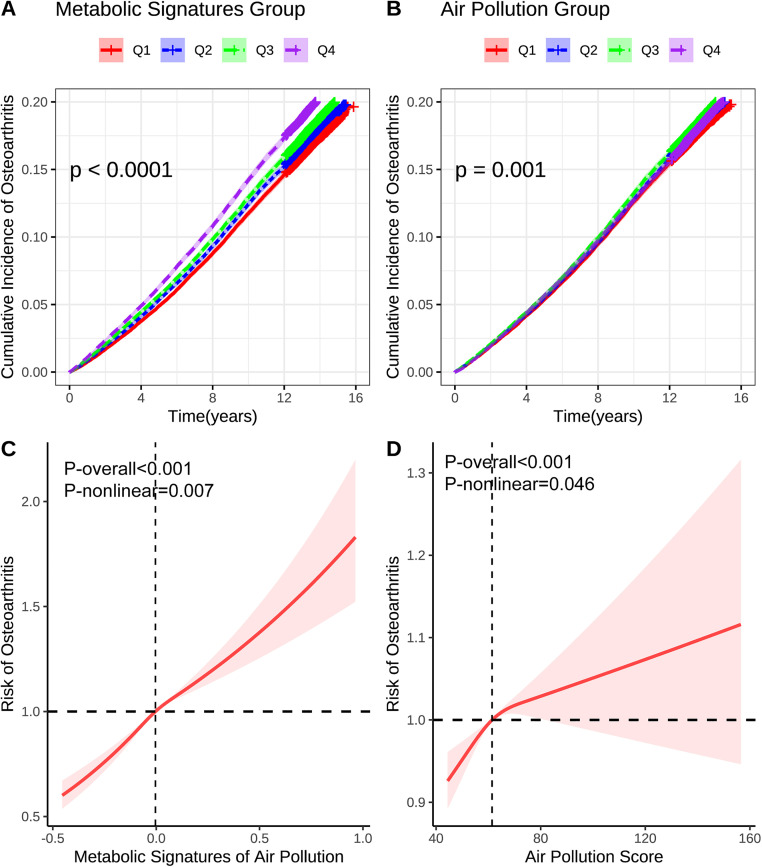
KM and RCS analysis of the associations of air pollution and the related metabolic profiles with osteoarthritis. Models were adjusted for age, sex, race, education, income, physical activity, smoke, alcohol, DASH, BMI, history of diabetes mellitus, hypertension, CVD, and cancer. BMI: Body mass index; DASH: Dietary approaches to stop hypertension; CVD: Cardiovascular disease.

**Fig 3:**
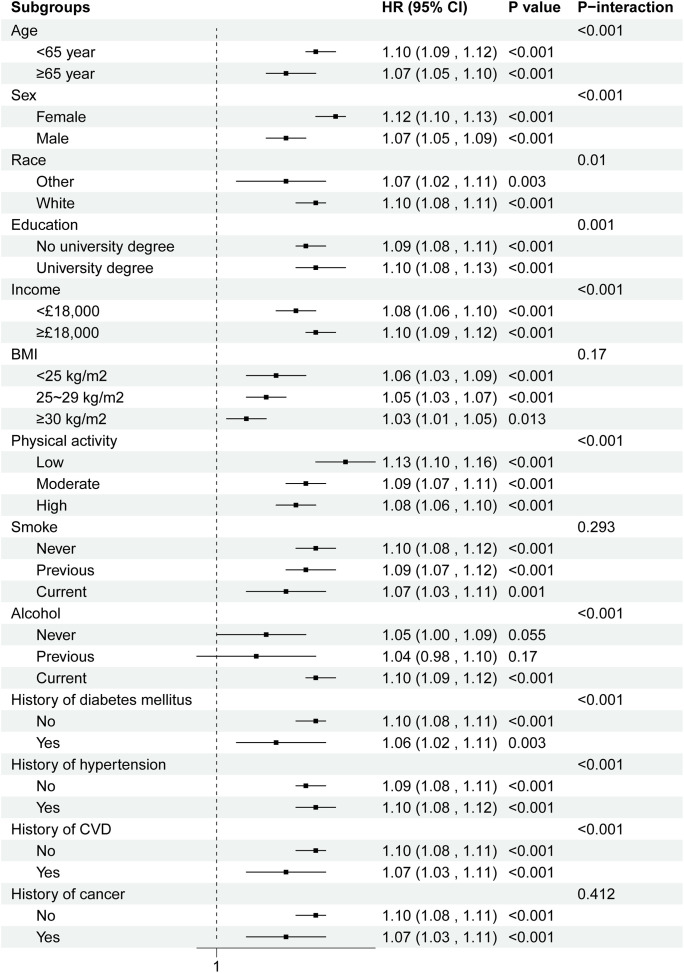
Subgroup analysis of the associations of the air pollution related metabolic profiles with osteoarthritis. Models were adjusted for age, sex, race, education, income, physical activity, smoke, alcohol, DASH, PRS, history of diabetes mellitus, hypertension, CVD, and cancer. BMI: Body mass index; DASH: Dietary approaches to stop hypertension; PRS: Polygenic risk score; CVD: Cardiovascular disease. The strata variable was not included in the model when stratifying by itself.

PM₂.₅, NO₂, and NOₓ showed significant positive associations with incident OA, with HR of 1.035 (95% CI: 1.023–1.048), 1.021 (95% CI: 1.007–1.034), and 1.025 (95% CI: 1.014–1.036) per IQR increase (sTable 2 in S1 File). We constructed an air pollution score based on the relationship between air pollution components and OA. Using elastic net regularized regression, we successfully identified 50 metabolic signatures associated with air pollution score (Fig 1, sTable 3 in S1 File). The identified metabolites encompassed diverse metabolic pathways (Fig 2), with relative lipoprotein lipid concentrations representing the largest category (26%), followed by fatty acids (16%), lipoprotein subclasses (14%), and amino acids (12%). Additional metabolite classes included glycolysis-related metabolites, ketone bodies, fluid balance markers, and inflammatory biomarkers, collectively capturing the multifaceted metabolic response to air pollution exposure. These metabolic signatures demonstrated strong correlations with their respective air pollutants (Fig 3). Importantly, participants who developed OA exhibited significantly higher metabolic signature scores compared to controls across all air pollution-related signatures (sTable 4 in S1 File).

### 3.3. Associations of air pollution and the related metabolic profiles with osteoarthritis

The air pollution-related metabolic signatures demonstrated stronger associations with incident OA compared to the composite air pollution score itself (Table 2). In the fully adjusted model (Model 3), each IQR increase in the metabolic profiles was associated with a 9.5% increased risk of developing OA (HR 1.095, 95% CI: 1.082–1.108), while the air pollution score showed a more modest 3.0% increased risk (HR 1.030, 95% CI: 1.018–1.042). Quartile analysis revealed pronounced dose-response relationships for both exposures, with participants in the highest metabolic profile quartile experiencing a 23.2% increased risk (HR 1.232, 95% CI: 1.197–1.268) compared to a 6.9% increased risk for the highest air pollution score quartile (HR 1.069, 95% CI: 1.039–1.100). KM survival curves demonstrated progressively higher cumulative incidence across increasing exposure quartiles (Fig 2A-B), while restricted cubic spline analysis confirmed significant non-linear relationships (Fig 2C-D).

**Table 2:** Associations of air pollution and related metabolic profiles with osteoarthritis.

Exposure	Model 1		Model 2		Model 3	
	HR(95%CI)	*P*	HR(95%CI)	*P*	HR(95%CI)	*P*
Metabolic profiles of air pollution						
Each IQR increment	1.131(1.118,1.144)	<0.0001	1.122(1.109,1.135)	<0.0001	1.095(1.082,1.108)	<0.0001
Q1	ref		ref		ref	
Q2	1.104(1.073,1.136)	<0.0001	1.097(1.066,1.129)	<0.0001	1.081(1.051,1.112)	<0.0001
Q3	1.176(1.143,1.209)	<0.0001	1.163(1.130,1.196)	<0.0001	1.128(1.097,1.161)	<0.0001
Q4	1.329(1.292,1.367)	<0.0001	1.303(1.267,1.340)	<0.0001	1.232(1.197,1.268)	<0.0001
P for trend		<0.0001		<0.0001		<0.0001
Air pollution score						
Each IQR increment	1.042(1.030,1.054)	<0.0001	1.035(1.023,1.047)	<0.0001	1.030(1.018,1.042)	<0.0001
Q1	ref		ref		ref	
Q2	1.023(0.995,1.051)	0.116	1.019(0.991,1.048)	0.187	1.014(0.986,1.042)	0.338
Q3	1.067(1.038,1.097)	<0.0001	1.060(1.031,1.090)	<0.0001	1.051(1.023,1.081)	<0.001
Q4	1.096(1.065,1.127)	<0.0001	1.081(1.051,1.112)	<0.0001	1.069(1.039,1.100)	<0.0001
P for trend		<0.0001		<0.0001		<0.0001

Model 1 was adjusted for age, sex, race, education, and income;

Model 2 was adjusted for Model 1 + physical activity, smoke, alcohol, DASH, and BMI;

Model 3 was adjusted for Model 2 + history of diabetes mellitus, hypertension, CVD, and cancer.

BMI: Body mass index; DASH: Dietary approaches to stop hypertension; CVD: Cardiovascular disease.

Site-specific analyses revealed differential associations across OA subtypes, with the strongest effects observed for knee OA (HR 1.140 per IQR increase, 95% CI: 1.118–1.162) (Table 3). Among individual metabolites, several demonstrated significant independent associations with OA risk (sTable 5 in S1 File), including decreased levels of albumin (HR 0.940, 95% CI: 0.931–0.950), HDL size (HR 0.905, 95% CI: 0.895–0.915), and increased levels of glycoprotein acetyls (HR 1.109, 95% CI: 1.099–1.120), reflecting inflammation and metabolic dysfunction pathways. Subgroup analyses indicated consistent associations across demographic strata, with significant interactions observed for age, sex, education, and comorbidity status (Fig 3).

**Table 3:** Associations of air pollution related metabolic profiles with subset of osteoarthritis.

Outcome	Model 1		Model 2		Model 3	
	HR(95%CI)	*P*	HR(95%CI)	*P*	HR(95%CI)	*P*
Knee osteoarthritis						
Each IQR increment	1.162(1.141,1.183)	<0.0001	1.163(1.142,1.184)	<0.0001	1.140(1.118,1.162)	<0.0001
Q1	ref		ref		ref	
Q2	1.156(1.102,1.212)	<0.0001	1.155(1.101,1.211)	<0.0001	1.139(1.086,1.195)	<0.0001
Q3	1.258(1.201,1.319)	<0.0001	1.257(1.199,1.318)	<0.0001	1.223(1.167,1.283)	<0.0001
Q4	1.442(1.376,1.511)	<0.0001	1.442(1.376,1.511)	<0.0001	1.373(1.309,1.441)	<0.0001
P for trend		<0.0001		<0.0001		<0.0001
Hip osteoarthritis						
Each IQR increment	0.997(0.971,1.023)	0.816	0.999(0.973,1.026)	0.944	0.994(0.968,1.021)	0.664
Q1	ref		ref		ref	
Q2	1.024(0.968,1.083)	0.404	1.025(0.969,1.084)	0.394	1.020(0.965,1.080)	0.481
Q3	0.996(0.940,1.054)	0.882	0.997(0.941,1.056)	0.915	0.989(0.933,1.047)	0.696
Q4	0.995(0.939,1.054)	0.864	0.999(0.942,1.059)	0.962	0.987(0.930,1.048)	0.671
P for trend		0.651		0.745		0.472
Hand osteoarthritis						
Each IQR increment	1.150(0.962,1.373)	0.125	1.160(0.970,1.387)	0.103	1.139(0.946,1.372)	0.169
Q1	ref		ref		ref	
Q2	0.812(0.519,1.270)	0.361	0.816(0.521,1.276)	0.372	0.807(0.516,1.263)	0.349
Q3	1.153(0.763,1.741)	0.499	1.169(0.773,1.768)	0.459	1.144(0.755,1.732)	0.526
Q4	1.349(0.900,2.022)	0.148	1.376(0.914,2.071)	0.126	1.326(0.874,2.012)	0.184
P for trend		0.058		0.048		0.079

### 3.4. Mediation of metabolic signature on the association of air pollution with osteoarthritis

**Fig 4:**
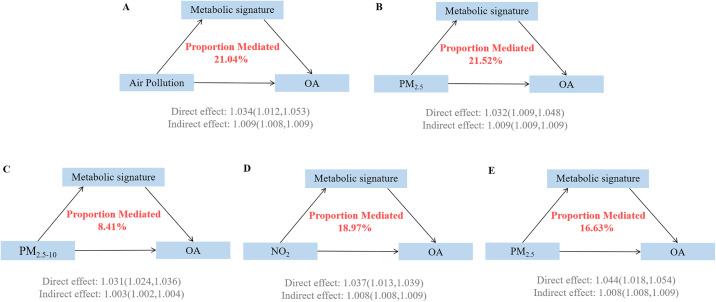
Mediation proportion of metabolic signature on the association of air pollution related metabolic profiles with osteoarthritis. Models were adjusted for age, sex, race, education, income, physical activity, smoke, alcohol, DASH, BMI, history of diabetes mellitus, hypertension, CVD, and cancer. BMI: Body mass index; DASH: Dietary approaches to stop hypertension; CVD: Cardiovascular disease.

Causal mediation analysis revealed that the air pollution-related metabolic signatures significantly mediated the associations between air pollution exposure and OA risk (Fig 4, sTable 6 in S1 File). For the composite air pollution score, the metabolic signature mediated 21.04% (95% CI: 16.52%−41.95%) of the total effect on OA development (P < 0.001). The mediation proportions varied across individual air pollutants, with PM₂.₅ showing the highest mediation percentage at 21.52% (95% CI: 16.73%−48.43%), followed by NOₓ at 16.63% (95% CI: 15.28%−32.26%), NO₂ at 18.97% (95% CI: 18.91%−39.07%). And PM₂.₅ ₋ ₁₀ at 8.41% (95% CI: 5.78%−10.83%).

Analysis of individual metabolites revealed differential mediation patterns (sTable 7 in S1 File). Several key metabolites showed significant mediation effects, including albumin (7.43% mediation, 95% CI: 7.57%−34.94%), HDL size (10.12% mediation, 95% CI: 10.94%−56.85%), and glycoprotein acetyls (6.28% mediation, 95% CI: 4.85%−32.52%).

### 3.5. Sensitive analysis

Multiple sensitivity analyses confirmed the robustness of our findings (sTables 8–13 in S1 File). Complete case analysis excluding participants with missing covariates demonstrated consistent associations between metabolic profiles and OA risk (HR 1.091, 95% CI: 1.073–1.109), with similar mediation proportions (29.14%). Exclusion of participants developing OA within two years of follow-up to address reverse causation yielded slightly attenuated but significant associations (HR 1.088, 95% CI: 1.074–1.103), maintaining substantial mediation effects (18.79%). Analysis restricted to participants without baseline chronic diseases (diabetes, hypertension, CVD, or cancer) continued to show significant associations (HR 1.093, 95% CI: 1.074–1.113) and meaningful mediation proportions (23.87%).

## 4. Discussion

This large-scale prospective cohort study provides the first comprehensive evidence that metabolic pathways mediate the air pollution-OA association. Using advanced metabolomics profiling and machine learning approaches, we identified distinct metabolic signatures comprising 50 air pollution-related metabolites that captured the biological response to environmental exposure. These signatures demonstrated significant prospective associations with incident OA risk. Causal mediation analysis revealed that metabolic alterations mediated approximately 21% of the air pollution-OA association, indicating that pollution-induced metabolic dysfunction represents a key mechanistic pathway. Associations were most pronounced for knee OA. These findings advance understanding of biological mechanisms linking environmental exposures to musculoskeletal health and identify metabolic biomarkers for risk stratification and targeted OA prevention strategies.

Numerous investigations have systematically evaluated air pollution-related metabolomic signatures, providing a robust foundation for comparing our findings. A comprehensive review by Liang et al.[13] synthesized 47 untargeted metabolomics studies and identified 816 unique metabolomic features associated with individual air pollutants, with oxidative stress and inflammation pathways being most commonly perturbed. Recent large-scale investigations have typically focused on single pollutant effects, with Wang et al.[16] and Ran et al. [21] reporting 65–87 metabolites for individual air pollutants (PM₂.₅, PM₁₀, NO₂, NOₓ) in pulmonary fibrosis and liver disease research. Similarly, parallel UK Biobank studies including Yang et al. [15] and Zhuo et al. [14] identified 85–106 metabolites associated with individual pollutant exposures in COPD and chronic respiratory disease research. In contrast to these single-pollutant approaches, our study represents a methodological advancement by constructing metabolic signatures for a composite air pollution exposure score that captures the integrated biological response to multiple pollutants simultaneously. Our identification of 50 metabolites associated with this comprehensive air pollution score encompasses lipoprotein subclasses, fatty acids, and amino acids, reflecting the complex multi-pollutant exposure reality. The metabolic pathways identified align with controlled exposure studies by Wang et al. [22] demonstrating individual pollutant effects on lipid and amino acid metabolism. These concordant observations support the biological plausibility of our composite approach and suggest that integrated air pollution metrics may better capture the cumulative metabolomic perturbations underlying chronic disease pathogenesis.

Our findings demonstrate remarkable consistency with previous OA metabolomics research while introducing significant methodological innovations. The 50 air pollution-related metabolites identified align substantially with established OA metabolomic signatures. Anderson et al. [23] revealed altered glycolysis and tricarboxylic acid cycle metabolites in OA synovial fluid through ¹H NMR spectroscopy, directly supporting our identification of glycolysis-related metabolites and energy metabolism pathway disruptions. Ge et al. [24] further confirmed upregulated tricarboxylic acid cycles, abnormal arginine metabolism, and collagen degradation in OA, validating our finding that amino acids (12%) and lipoprotein subclasses (14%) comprise significant proportions of air pollution-related metabolic signatures. Yang et al. [25] demonstrated through Mendelian randomization that elevated lactate levels associate with increased OA risk and conjugated linoleic acid ratios affect multiple OA phenotypes, consistent with our identified fatty acid metabolism pathways (16%) and inflammatory biomarkers. Unlike Mickiewicz et al.[26] and Akhbari et al. [27] who focused on localized synovial fluid changes, we pioneered the construction of comprehensive air pollution scores capturing circulation metabolic signatures in large-scale population cohorts, quantifying metabolic pathway mediation in environment-disease mechanisms through causal mediation analysis. While Li et al. [28] identified lipid metabolism biomarkers through machine learning at gene expression levels, our research directly captured environmental exposure effects at circulating metabolite levels, providing more direct mechanistic evidence for understanding how environmental factors influence joint health through metabolic remodeling.

The mechanistic pathway linking air pollution to OA development operates through systematic metabolic reprogramming across. Air pollution exposure fundamentally disrupts cellular energy metabolism by impairing mitochondrial function and altering key metabolic enzyme activities. Ginos et al.[29] demonstrated that long-term PM₂.₅ and nitrogen dioxide exposure significantly altered lipid and amino acid subclasses, while Reyes-Caballero et al.[30] revealed that particulate matter compromises hepatic glucose metabolism, reducing glycolytic intermediates and Krebs cycle capacity while enhancing fatty acid synthesis pathways. These systemic metabolic perturbations subsequently cascade into joint tissues, triggering profound chondrocyte dysfunction through several critical mechanisms. First, pollution-induced alterations in cholesterol metabolism activate the CH25H-CYP7B1-RORα axis, leading to increased cholesterol uptake and oxysterol production that directly promotes cartilage matrix degradation [31]. Second, the metabolic shift toward enhanced glycolysis and impaired oxidative phosphorylation, compromises chondrocyte energy homeostasis, forcing cells into a catabolic state [32,33]. Third, mitochondrial dysfunction disrupts the SIRT3-PINK1-PKM2 axis, impairing mitochondrial renewal and metabolic switching capabilities essential for cartilage maintenance [34]. Fourth, lipid metabolism disorders create a pro-inflammatory microenvironment that amplifies joint destruction through enhanced inflammatory cytokine production [35,36]. Finally, amino acid metabolism dysregulation compromises collagen synthesis while promoting protein degradation [37,38].

Our findings carry profound implications for clinical practice. The identified 50 metabolites, particularly albumin, HDL size, and glycoprotein acetyls, could serve as accessible biomarkers for early risk stratification before irreversible joint damage occurs. The substantial mediation effect (21%) suggests that metabolic interventions could meaningfully reduce air pollution-associated OA risk. Henry & O’Neill [39] emphasized that targeting metabolic reprogramming represents a promising therapeutic strategy for connective tissue disorders, while Chen et al. [40] demonstrated that regulating lipid metabolism shows important therapeutic potential in OA management.

Several limitations warrant consideration in our findings. First, the observational design precludes definitive causal inference despite our comprehensive mediation analysis and sensitivity testing. Second, air pollution exposure assessment relied on residential address modeling rather than personal monitoring, potentially introducing exposure misclassification. Third, metabolomic profiling was conducted at baseline only, preventing assessment of temporal metabolic changes or dose-response relationships over time. Fourth, the UK Biobank population consists predominantly of middle-aged, educated, white Europeans, potentially limiting generalizability to younger, more diverse populations with different pollution exposure patterns. Fifth, residual confounding from unmeasured lifestyle factors, genetic variants, or occupational exposures cannot be entirely excluded. Finally, the NMR metabolomics platform, while comprehensive, captures only a subset of the complete metabolome and may miss other relevant metabolic pathways.

## 5. Conclusion

This large-scale prospective cohort study provides the evidence that metabolic pathways significantly mediate the association between air pollution exposure and OA development. We identified 50 air pollution-related metabolites spanning lipoprotein metabolism, fatty acid pathways, and inflammatory responses, which demonstrated stronger associations with incident OA than pollution exposure alone. Causal mediation analysis revealed that metabolic alterations account for approximately 21% of the pollution-OA relationship, elucidating specific biological mechanisms linking atmospheric pollutants to joint disease through systematic metabolic reprogramming.

## Supporting information

S1 File Supplement. (DOCX)
