# Supplementary material for: Metabolic pathways linking air pollution to osteoarthritis: Insights from a prospective cohort
Source: PLoS One. 2026 Jan 20;21(1):e0341125. doi: 10.1371/journal.pone.0341125 (PMC12818614; doi:10.1371/journal.pone.0341125)
Supplement: S1 File — (DOCX) [file pone.0341125.s001.docx]

**Metabolic Pathways Linking Air Pollution to Osteoarthritis: Insights from a Prospective Cohort**

Supplement

[sFigure 1 Flow chart for study population selection 3](#_Toc214441690)

[sFigure 2 Classification of metabolic signature of air pollution screened by elastic network regression 3](#_Toc214441691)

[sFigure 3 Correlation of the metabolic signature consisting of selected metabolites with air pollution and components. 4](#_Toc214441692)

[sFigure 4 RCS analysis of the associations between air pollution and the related metabolic profiles. 5](#_Toc214441693)

[sTable 1 ICD coding for osteoarthritis 6](#_Toc214441694)

[sTable 2 Age distribution of osteoarthritis and the control population 9](#_Toc214441695)

[sTable 3 Associations of air pollution with osteoarthritis 9](#_Toc214441696)

[sTable 4 Coefficients of air pollution metabolic signatures established by elastic network regression 10](#_Toc214441697)

[sTable 5 Median and Interquartile range of concentrations for the metabolites quantified by NMR 12](#_Toc214441698)

[sTable 6 Cox regression model to assess associations between metabolic biomarkers of air pollution and osteoarthritis 13](#_Toc214441699)

[sTable 7 Mediation of metabolic signature on the association of air pollution with OA 14](#_Toc214441700)

[sTable 8 Mediation proportion of air pollution metabolic biomarkers on the association of air pollution with osteoarthritis. 15](#_Toc214441701)

[sTable 9 Sensitive analysis of the associations of air pollution and the related metabolic profiles with osteoarthritis after exclusion of participants with missing covariates. 17](#_Toc214441702)

[sTable 10 Sensitive analysis of the mediation of metabolic signature on the association of air pollution with OA after exclusion of participants with missing covariates 17](#_Toc214441703)

[sTable 11 Sensitive analysis of the associations of air pollution and the related metabolic profiles with osteoarthritis after exclusion of participants who developed osteoarthritis within 2 years of follow-up. 18](#_Toc214441704)

[sTable 12 Sensitive analysis of the mediation of metabolic signature on the association of air pollution with OA after exclusion of participants who developed osteoarthritis within 2 years of follow-up 18](#_Toc214441705)

[sTable 13 Sensitive analysis of the associations of air pollution and the related metabolic profiles with osteoarthritis after exclusion of participants who has the history of chronic disease. 19](#_Toc214441706)

[sTable 14 Sensitive analysis of the mediation of metabolic signature on the association of air pollution with OA after exclusion of participants who has the history of chronic disease. 20](#_Toc214441707)

[sTable 15 Sensitive analysis of the associations of air pollution and the related metabolic profiles with osteoarthritis after further adjustment of sleep duration. 21](#_Toc214441708)

[sTable 16 Sensitive analysis of the mediation of metabolic signature on the association of air pollution with OA after further adjustment of sleep duration. 21](#_Toc214441709)


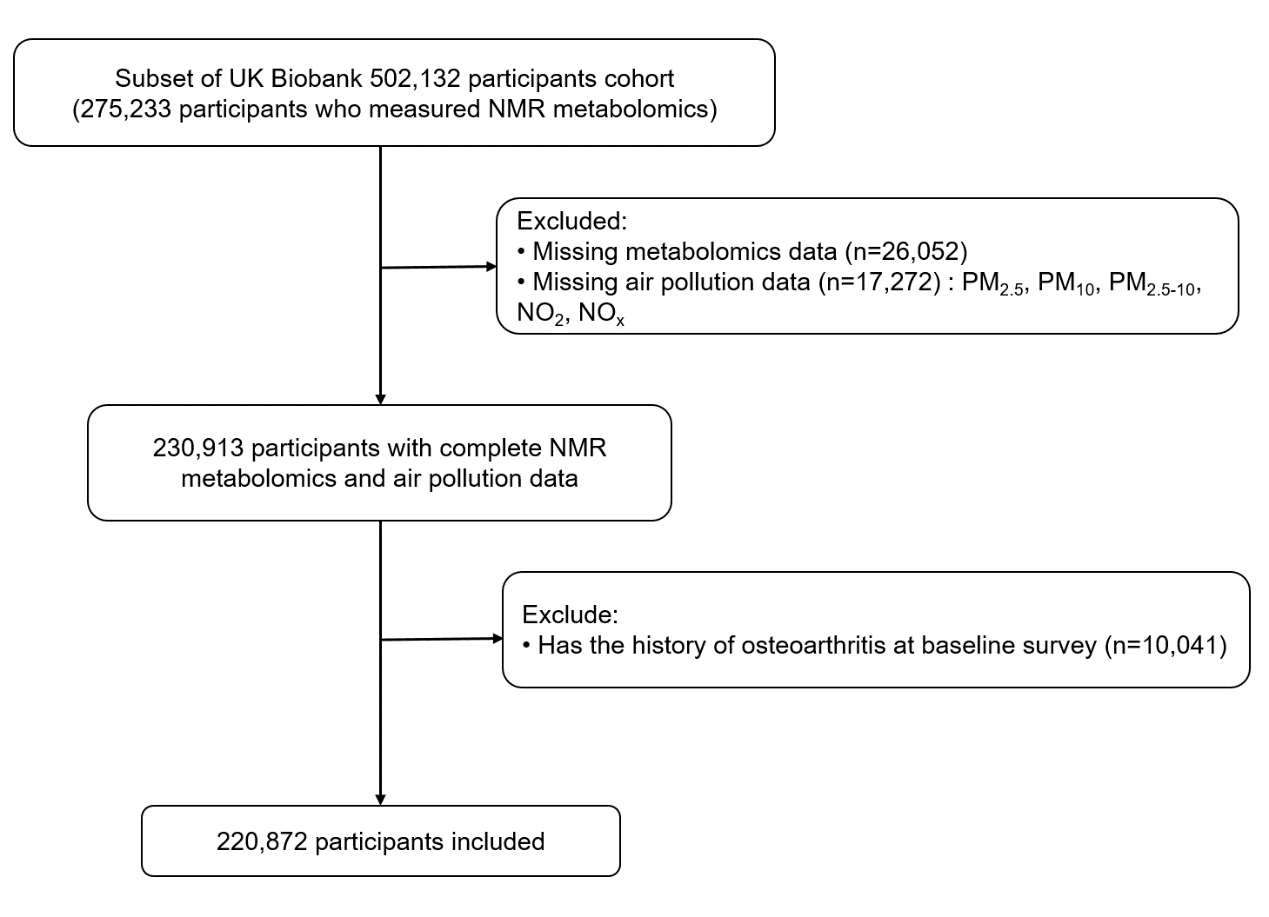
sFigure 1 Flow chart for study population selection

NMR: Nuclear magnetic resonance; BMI: Body mass index.


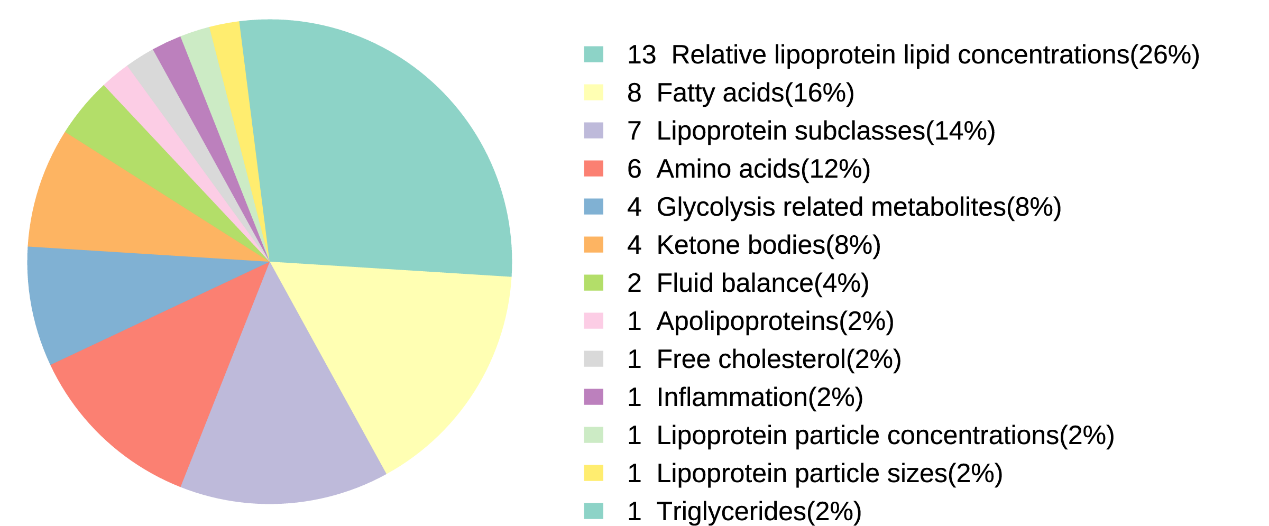


sFigure 2 Classification of metabolic signature of air pollution screened by elastic network regression


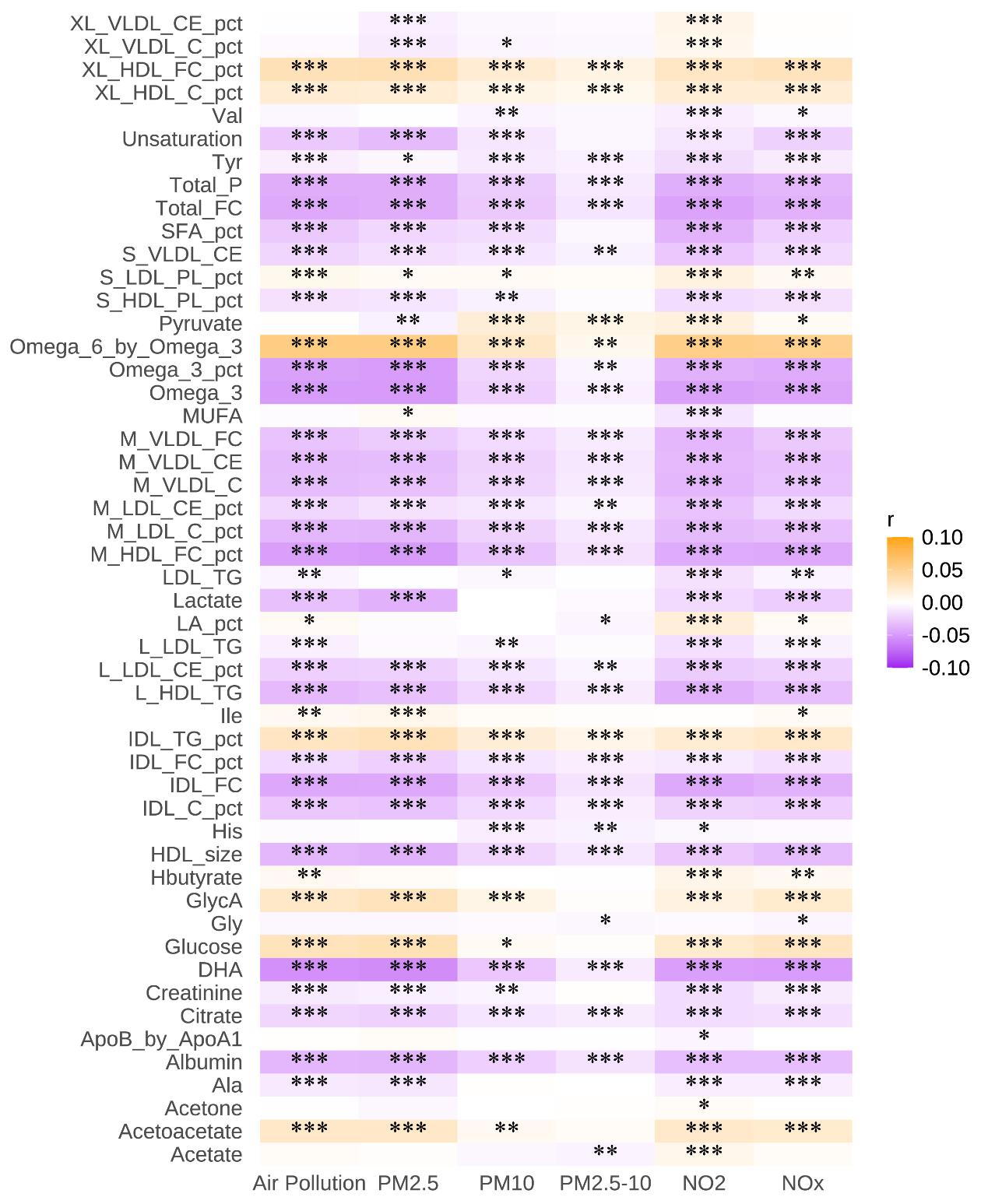


sFigure 3 Correlation of the metabolic signature consisting of selected metabolites with air pollution and components.

****P*<0.001, ***P*<0.01, **P*<0.05.


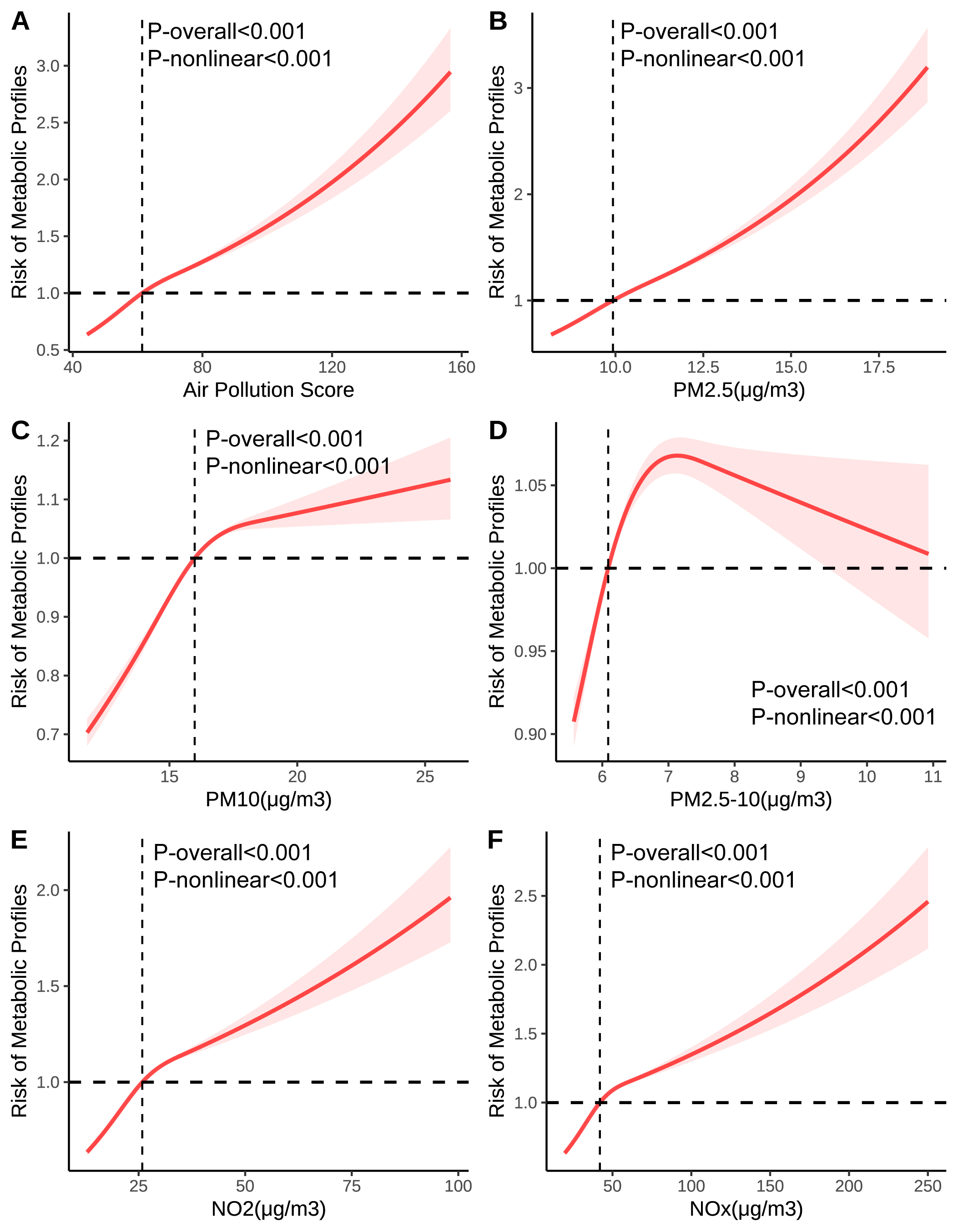


sFigure 4 RCS analysis of the associations between air pollution and the related metabolic profiles.

sTable 1 ICD coding for osteoarthritis

| **OA** |  | **Knee OA** |  | **Hip OA** |  | **Hand OA** |
| --- | --- | --- | --- | --- | --- | --- |
| **ICD10** | **ICD9** | **ICD10** | **ICD9** | **ICD10** | **ICD9** | **ICD10** |
| M15.0 | 715 | M17 | 71536 | M16 | 71535 | M19.041 |
| M15.1 | 7150 | M17.0 | 71516 | M16.0 | 71515 | M19.042 |
| M15.2 | 7151 | M17.1 |  | M16.1 |  | M19.04 |
| M15.99 | 71510 | M17.9 |  | M16.9 |  | M19.049 |
| M16 | 71511 | M19.06 |  | M19.05 |  | M18.0 |
| M16.0 | 71512 | M19.96 |  | M19.95 |  | M18.1 |
| M16.1 | 71513 |  |  |  |  | M18.10 |
| M16.9 | 71514 |  |  |  |  | M18.11 |
| M17 | 71515 |  |  |  |  | M18.12 |
| M17.0 | 71516 |  |  |  |  | M18.9 |
| M17.1 | 71517 |  |  |  |  |  |
| M17.9 | 71518 |  |  |  |  |  |
| M18 | 71519 |  |  |  |  |  |
| M18.0 | 7152 |  |  |  |  |  |
| M18.1 | 71520 |  |  |  |  |  |
| M18.9 | 71521 |  |  |  |  |  |
| M19 | 71522 |  |  |  |  |  |
| M19.0 | 71523 |  |  |  |  |  |
| M19.00 | 71524 |  |  |  |  |  |
| M19.01 | 71525 |  |  |  |  |  |
| M19.02 | 71526 |  |  |  |  |  |
| M19.03 | 71527 |  |  |  |  |  |
| M19.04 | 71528 |  |  |  |  |  |
| M19.05 | 71529 |  |  |  |  |  |
| M19.06 | 7153 |  |  |  |  |  |
| M19.07 | 71530 |  |  |  |  |  |
| M19.08 | 71531 |  |  |  |  |  |
| M19.09 | 71532 |  |  |  |  |  |
| M19.9 | 71533 |  |  |  |  |  |
| M19.90 | 71534 |  |  |  |  |  |
| M19.91 | 71535 |  |  |  |  |  |
| M19.92 | 71536 |  |  |  |  |  |
| M19.93 | 71537 |  |  |  |  |  |
| M19.94 | 71538 |  |  |  |  |  |
| M19.95 | 71539 |  |  |  |  |  |
| M19.96 | 7158 |  |  |  |  |  |
| M19.97 | 7159 |  |  |  |  |  |
| M19.98 | 721 |  |  |  |  |  |
| M19.99 | 7210 |  |  |  |  |  |
| M47.2 | 7211 |  |  |  |  |  |
| M47.20 | 7212 |  |  |  |  |  |
| M47.21 | 7213 |  |  |  |  |  |
| M47.22 | 7214 |  |  |  |  |  |
| M47.23 | 7215 |  |  |  |  |  |
| M47.24 | 7216 |  |  |  |  |  |
| M47.25 | 7217 |  |  |  |  |  |
| M47.26 | 7218 |  |  |  |  |  |
| M47.27 | 7219 |  |  |  |  |  |
| M47.28 |  |  |  |  |  |  |
| M47.29 |  |  |  |  |  |  |
| M47.8 |  |  |  |  |  |  |
| M47.80 |  |  |  |  |  |  |
| M47.81 |  |  |  |  |  |  |
| M47.82 |  |  |  |  |  |  |
| M47.83 |  |  |  |  |  |  |
| M47.84 |  |  |  |  |  |  |
| M47.85 |  |  |  |  |  |  |
| M47.86 |  |  |  |  |  |  |
| M47.87 |  |  |  |  |  |  |
| M47.88 |  |  |  |  |  |  |
| M47.89 |  |  |  |  |  |  |
| M47.9 |  |  |  |  |  |  |
| M47.90 |  |  |  |  |  |  |
| M47.91 |  |  |  |  |  |  |
| M47.92 |  |  |  |  |  |  |
| M47.93 |  |  |  |  |  |  |
| M47.94 |  |  |  |  |  |  |
| M47.95 |  |  |  |  |  |  |
| M47.96 |  |  |  |  |  |  |
| M47.97 |  |  |  |  |  |  |
| M47.98 |  |  |  |  |  |  |

sTable 2 Age distribution of osteoarthritis and the control population

| Level | Overall  (*n*= 220872) | Control  (*n*= 180473) | Osteoarthritis  (*n*= 40399) | *P* |
| --- | --- | --- | --- | --- |
| Age (years) |  |  |  | <0.001 |
| <45 | 23090 (10.45) | 21416 (11.87) | 1674 ( 4.14) |  |
| 46-49 | 29322 (13.28) | 26468 (14.67) | 2854 ( 7.06) |  |
| 50-54 | 33664 (15.24) | 28964 (16.05) | 4700 (11.63) |  |
| 55-59 | 39849 (18.04) | 32656 (18.09) | 7193 (17.80) |  |
| 60-64 | 53788 (24.35) | 41434 (22.96) | 12354 (30.58) |  |
| ≥65 | 41159 (18.63) | 29535 (16.37) | 11624 (28.77) |  |

sTable 3 Associations of air pollution with osteoarthritis

| Outcome | Model 1 | | Model 2 | | Model 3 | |
| --- | --- | --- | --- | --- | --- | --- |
|  | HR(95%CI) | *P* | HR(95%CI) | *P* | HR(95%CI) | *P* |
| PM25 | | | | | | |
| Each IQR increment | 1.046(1.034,1.059) | <0.0001 | 1.040(1.028,1.053) | <0.0001 | 1.035(1.023,1.048) | <0.0001 |
| Q1 | ref |  | ref |  | ref |  |
| Q2 | 1.022(0.994,1.050) | 0.132 | 1.018(0.990,1.047) | 0.211 | 1.013(0.985,1.041) | 0.381 |
| Q3 | 1.057(1.028,1.087) | <0.0001 | 1.051(1.022,1.081) | <0.001 | 1.044(1.015,1.073) | 0.003 |
| Q4 | 1.099(1.068,1.130) | <0.0001 | 1.085(1.054,1.115) | <0.0001 | 1.073(1.043,1.103) | <0.0001 |
| P for trend |  | <0.0001 |  | <0.0001 |  | <0.0001 |
| PM10 |  |  |  |  |  |  |
| Each IQR increment | 1.005(0.996,1.014) | 0.315 | 1.002(0.993,1.011) | 0.618 | 1.000(0.991,1.009) | 0.969 |
| Q1 | ref |  | ref |  | ref |  |
| Q2 | 1.034(1.006,1.063) | 0.016 | 1.031(1.003,1.060) | 0.028 | 1.029(1.001,1.058) | 0.040 |
| Q3 | 1.031(1.003,1.060) | 0.031 | 1.024(0.996,1.053) | 0.087 | 1.021(0.993,1.049) | 0.151 |
| Q4 | 1.014(0.986,1.042) | 0.344 | 1.006(0.978,1.035) | 0.670 | 1.001(0.973,1.029) | 0.960 |
| P for trend |  | 0.399 |  | 0.787 |  | 0.897 |
| PM25-10 |  |  |  |  |  |  |
| Each IQR increment | 1.002(0.993,1.010) | 0.710 | 1.000(0.992,1.009) | 0.965 | 0.999(0.991,1.007) | 0.843 |
| Q1 | ref |  | ref |  | ref |  |
| Q2 | 1.052(1.023,1.081) | <0.001 | 1.049(1.020,1.078) | <0.001 | 1.045(1.017,1.074) | 0.002 |
| Q3 | 1.045(1.016,1.074) | 0.002 | 1.039(1.011,1.068) | 0.007 | 1.035(1.007,1.064) | 0.015 |
| Q4 | 1.035(1.006,1.064) | 0.016 | 1.028(1.000,1.057) | 0.052 | 1.022(0.994,1.051) | 0.118 |
| P for trend |  | 0.03 |  | 0.094 |  | 0.197 |
| NO2 |  |  |  |  |  |  |
| Each IQR increment | 1.033(1.020,1.047) | <0.0001 | 1.026(1.013,1.039) | <0.001 | 1.021(1.007,1.034) | 0.002 |
| Q1 | ref |  | ref |  | ref |  |
| Q2 | 1.047(1.018,1.076) | 0.001 | 1.043(1.015,1.073) | 0.003 | 1.036(1.008,1.066) | 0.011 |
| Q3 | 1.068(1.039,1.098) | <0.0001 | 1.061(1.032,1.091) | <0.0001 | 1.052(1.023,1.082) | <0.001 |
| Q4 | 1.075(1.045,1.106) | <0.0001 | 1.060(1.030,1.091) | <0.0001 | 1.049(1.019,1.079) | 0.001 |
| P for trend |  | <0.0001 |  | <0.0001 |  | <0.001 |
| NOx |  |  |  |  |  |  |
| Each IQR increment | 1.035(1.024,1.045) | <0.0001 | 1.029(1.018,1.040) | <0.0001 | 1.025(1.014,1.036) | <0.0001 |
| Q1 | ref |  | ref |  | ref |  |
| Q2 | 1.035(1.007,1.064) | 0.015 | 1.032(1.003,1.061) | 0.028 | 1.026(0.998,1.055) | 0.066 |
| Q3 | 1.078(1.049,1.109) | <0.0001 | 1.071(1.042,1.101) | <0.0001 | 1.063(1.034,1.093) | <0.0001 |
| Q4 | 1.101(1.071,1.133) | <0.0001 | 1.086(1.056,1.118) | <0.0001 | 1.074(1.044,1.105) | <0.0001 |
| P for trend |  | <0.0001 |  | <0.0001 |  | <0.0001 |

Model 1 was adjusted for age, sex, race, education, and income;

Model 2 was adjusted for Model 1 + physical activity, smoke, alcohol, DASH and BMI;

Model 3 was adjusted for Model 2 + history of diabetes mellitus, hypertension, CVD, and cancer.

BMI: Body mass index; DASH: Dietary approaches to stop hypertension; CVD: Cardiovascular disease.

sTable 4 Coefficients of air pollution metabolic signatures established by elastic network regression

| No. | Metabolite | Coefficient | Full name of Metabolite | Group |
| --- | --- | --- | --- | --- |
| 1 | Hbutyrate | -0.0002 | 3-Hydroxybutyrate | Ketone bodies |
| 2 | Acetate | 0.0035 | Acetate | Ketone bodies |
| 3 | Acetoacetate | 0.0258 | Acetoacetate | Ketone bodies |
| 4 | Acetone | -0.0175 | Acetone | Ketone bodies |
| 5 | Ala | -0.0005 | Alanine | Amino acids |
| 6 | Albumin | -0.0102 | Albumin | Fluid balance |
| 7 | ApoB_by_ApoA1 | 0.0079 | Apolipoprotein B to Apolipoprotein A1 ratio | Apolipoproteins |
| 8 | HDL_size | -0.0119 | Average Diameter for HDL Particles | Lipoprotein particle sizes |
| 9 | M_VLDL_C | -0.0242 | Cholesterol in Medium VLDL | Lipoprotein subclasses |
| 10 | IDL_C_pct | -0.0033 | Cholesterol to Total Lipids in IDL percentage | Relative lipoprotein lipid concentrations |
| 11 | M_LDL_C_pct | -0.0063 | Cholesterol to Total Lipids in Medium LDL percentage | Relative lipoprotein lipid concentrations |
| 12 | XL_HDL_C_pct | 0.0011 | Cholesterol to Total Lipids in Very Large HDL percentage | Relative lipoprotein lipid concentrations |
| 13 | XL_VLDL_C_pct | 0.0001 | Cholesterol to Total Lipids in Very Large VLDL percentage | Relative lipoprotein lipid concentrations |
| 14 | M_VLDL_CE | -0.0125 | Cholesteryl Esters in Medium VLDL | Lipoprotein subclasses |
| 15 | S_VLDL_CE | -0.0008 | Cholesteryl Esters in Small VLDL | Lipoprotein subclasses |
| 16 | L_LDL_CE_pct | -0.0045 | Cholesteryl Esters to Total Lipids in Large LDL percentage | Relative lipoprotein lipid concentrations |
| 17 | M_LDL_CE_pct | -0.0109 | Cholesteryl Esters to Total Lipids in Medium LDL percentage | Relative lipoprotein lipid concentrations |
| 18 | XL_VLDL_CE_pct | 0.0151 | Cholesteryl Esters to Total Lipids in Very Large VLDL percentage | Relative lipoprotein lipid concentrations |
| 19 | Citrate | -0.015 | Citrate | Glycolysis related metabolites |
| 20 | Creatinine | -0.0181 | Creatinine | Fluid balance |
| 21 | Unsaturation | 0.0403 | Degree of Unsaturation | Fatty acids |
| 22 | DHA | -0.0117 | Docosahexaenoic Acid | Fatty acids |
| 23 | IDL_FC | -0.0016 | Free Cholesterol in IDL | Lipoprotein subclasses |
| 24 | M_VLDL_FC | -0.0011 | Free Cholesterol in Medium VLDL | Lipoprotein subclasses |
| 25 | IDL_FC_pct | -0.0037 | Free Cholesterol to Total Lipids in IDL percentage | Relative lipoprotein lipid concentrations |
| 26 | M_HDL_FC_pct | -0.006 | Free Cholesterol to Total Lipids in Medium HDL percentage | Relative lipoprotein lipid concentrations |
| 27 | XL_HDL_FC_pct | 0.0051 | Free Cholesterol to Total Lipids in Very Large HDL percentage | Relative lipoprotein lipid concentrations |
| 28 | Glucose | 0.0164 | Glucose | Glycolysis related metabolites |
| 29 | Gly | 0.0032 | Glycine | Amino acids |
| 30 | GlycA | 0.0283 | Glycoprotein Acetyls | Inflammation |
| 31 | His | 0.0026 | Histidine | Amino acids |
| 32 | Ile | 0.0102 | Isoleucine | Amino acids |
| 33 | Lactate | -0.0325 | Lactate | Glycolysis related metabolites |
| 34 | LA_pct | -0.001 | Linoleic Acid to Total Fatty Acids percentage | Fatty acids |
| 35 | MUFA | 0.0202 | Monounsaturated Fatty Acids | Fatty acids |
| 36 | Omega_3 | -0.0151 | Omega-3 Fatty Acids | Fatty acids |
| 37 | Omega_3_pct | -0.0052 | Omega-3 Fatty Acids to Total Fatty Acids percentage | Fatty acids |
| 38 | Omega_6_by_Omega_3 | 0.0431 | Omega-6 Fatty Acids to Omega-3 Fatty Acids ratio | Fatty acids |
| 39 | S_HDL_PL_pct | 0.0001 | Phospholipids to Total Lipids in Small HDL percentage | Relative lipoprotein lipid concentrations |
| 40 | S_LDL_PL_pct | -0.0067 | Phospholipids to Total Lipids in Small LDL percentage | Relative lipoprotein lipid concentrations |
| 41 | Pyruvate | 0.0194 | Pyruvate | Glycolysis related metabolites |
| 42 | SFA_pct | -0.0128 | Saturated Fatty Acids to Total Fatty Acids percentage | Fatty acids |
| 43 | Total_P | -0.0001 | Total Concentration of Lipoprotein Particles | Lipoprotein particle concentrations |
| 44 | Total_FC | -0.0004 | Total Free Cholesterol | Free cholesterol |
| 45 | LDL_TG | 0.0006 | Triglycerides in LDL | Triglycerides |
| 46 | L_HDL_TG | -0.0183 | Triglycerides in Large HDL | Lipoprotein subclasses |
| 47 | L_LDL_TG | 0.0291 | Triglycerides in Large LDL | Lipoprotein subclasses |
| 48 | IDL_TG_pct | 0.0046 | Triglycerides to Total Lipids in IDL percentage | Relative lipoprotein lipid concentrations |
| 49 | Tyr | -0.0024 | Tyrosine | Amino acids |
| 50 | Val | -0.0081 | Valine | Amino acids |

sTable 5 Median and Interquartile range of concentrations for the metabolites quantified by NMR

| No. | Metabolite | Overall  (*n*= 220872) | Control  (*n*= 180473) | Osteoarthritis  (*n*= 40399) |
| --- | --- | --- | --- | --- |
| 1 | Hbutyrate | 0.043[0.030,0.068] | 0.043[0.029,0.068] | 0.044[0.030,0.069] |
| 2 | Acetate | 0.015[0.012,0.019] | 0.015[0.012,0.019] | 0.015[0.011,0.019] |
| 3 | Acetoacetate | 0.010[0.006,0.016] | 0.010[0.006,0.016] | 0.010[0.007,0.016] |
| 4 | Acetone | 0.013[0.011,0.016] | 0.013[0.011,0.016] | 0.013[0.011,0.016] |
| 5 | Ala | 0.289[0.240,0.345] | 0.288[0.240,0.345] | 0.290[0.242,0.346] |
| 6 | Albumin | 39.389[37.316,41.455] | 39.463[37.398,41.537] | 39.037[36.952,41.080] |
| 7 | ApoB_by_ApoA1 | 0.586[0.483,0.705] | 0.586[0.483,0.706] | 0.583[0.481,0.700] |
| 8 | HDL_size | 9.598[9.484,9.753] | 9.600[9.485,9.758] | 9.592[9.483,9.735] |
| 9 | M_VLDL_C | 0.169[0.127,0.215] | 0.169[0.128,0.215] | 0.167[0.124,0.215] |
| 10 | IDL_C_pct | 68.164[66.131,69.758] | 68.238[66.254,69.799] | 67.781[65.599,69.555] |
| 11 | M_LDL_C_pct | 68.724[67.713,69.450] | 68.749[67.763,69.464] | 68.604[67.495,69.385] |
| 12 | XL_HDL_C_pct | 49.393[47.140,52.382] | 49.417[47.169,52.417] | 49.268[46.998,52.217] |
| 13 | XL_VLDL_C_pct | 27.782[24.082,32.460] | 27.950[24.222,32.657] | 27.059[23.497,31.524] |
| 14 | M_VLDL_CE | 0.091[0.066,0.118] | 0.092[0.067,0.118] | 0.089[0.063,0.117] |
| 15 | S_VLDL_CE | 0.097[0.077,0.120] | 0.097[0.076,0.120] | 0.099[0.078,0.122] |
| 16 | L_LDL_CE_pct | 52.777[51.968,53.491] | 52.783[51.978,53.495] | 52.752[51.920,53.477] |
| 17 | M_LDL_CE_pct | 49.298[48.106,50.409] | 49.276[48.081,50.389] | 49.399[48.212,50.503] |
| 18 | XL_VLDL_CE_pct | 15.984[12.985,19.845] | 16.115[13.100,20.000] | 15.414[12.514,19.088] |
| 19 | Citrate | 0.065[0.057,0.073] | 0.064[0.056,0.073] | 0.065[0.057,0.074] |
| 20 | Creatinine | 0.066[0.058,0.075] | 0.066[0.058,0.075] | 0.066[0.059,0.076] |
| 21 | Unsaturation | 1.356[1.305,1.405] | 1.357[1.306,1.406] | 1.351[1.299,1.402] |
| 22 | DHA | 0.224[0.180,0.277] | 0.223[0.179,0.276] | 0.227[0.182,0.282] |
| 23 | IDL_FC | 0.217[0.181,0.256] | 0.218[0.182,0.256] | 0.217[0.177,0.257] |
| 24 | M_VLDL_FC | 0.078[0.060,0.099] | 0.078[0.060,0.099] | 0.079[0.060,0.100] |
| 25 | IDL_FC_pct | 17.794[17.054,18.468] | 17.810[17.080,18.481] | 17.716[16.938,18.408] |
| 26 | M_HDL_FC_pct | 8.302[7.862,8.741] | 8.306[7.863,8.747] | 8.285[7.856,8.712] |
| 27 | XL_HDL_FC_pct | 14.686[12.775,16.931] | 14.665[12.751,16.917] | 14.772[12.886,16.991] |
| 28 | Glucose | 3.558[3.117,4.031] | 3.544[3.105,4.013] | 3.619[3.175,4.105] |
| 29 | Gly | 0.158[0.128,0.201] | 0.159[0.128,0.201] | 0.156[0.126,0.199] |
| 30 | GlycA | 0.806[0.733,0.887] | 0.801[0.729,0.882] | 0.827[0.754,0.907] |
| 31 | His | 0.065[0.058,0.072] | 0.065[0.059,0.072] | 0.064[0.058,0.072] |
| 32 | Ile | 0.048[0.039,0.060] | 0.048[0.039,0.060] | 0.049[0.040,0.061] |
| 33 | Lactate | 3.859[3.189,4.618] | 3.861[3.191,4.626] | 3.848[3.184,4.588] |
| 34 | LA_pct | 29.087[26.745,31.218] | 29.232[26.912,31.347] | 28.414[26.060,30.598] |
| 35 | MUFA | 2.793[2.353,3.356] | 2.770[2.334,3.330] | 2.894[2.444,3.472] |
| 36 | Omega_3 | 0.499[0.379,0.647] | 0.494[0.376,0.642] | 0.519[0.397,0.669] |
| 37 | Omega_3_pct | 4.158[3.338,5.136] | 4.140[3.322,5.118] | 4.243[3.409,5.216] |
| 38 | Omega_6_by_Omega_3 | 9.025[7.182,11.466] | 9.105[7.238,11.560] | 8.683[6.965,11.014] |
| 39 | S_HDL_PL_pct | 57.026[56.231,57.835] | 57.003[56.206,57.813] | 57.132[56.345,57.930] |
| 40 | S_LDL_PL_pct | 31.122[30.088,32.226] | 31.140[30.105,32.244] | 31.041[30.009,32.133] |
| 41 | Pyruvate | 0.079[0.063,0.096] | 0.078[0.063,0.096] | 0.079[0.064,0.096] |
| 42 | SFA_pct | 33.880[32.730,35.147] | 33.840[32.692,35.099] | 34.066[32.903,35.352] |
| 43 | Total_P | 0.017[0.015,0.018] | 0.017[0.015,0.018] | 0.017[0.015,0.019] |
| 44 | Total_FC | 1.259[1.085,1.442] | 1.259[1.087,1.440] | 1.262[1.077,1.450] |
| 45 | LDL_TG | 0.141[0.119,0.169] | 0.140[0.118,0.167] | 0.146[0.124,0.174] |
| 46 | L_HDL_TG | 0.029[0.022,0.037] | 0.028[0.022,0.037] | 0.030[0.023,0.038] |
| 47 | L_LDL_TG | 0.095[0.081,0.112] | 0.094[0.080,0.111] | 0.098[0.084,0.116] |
| 48 | IDL_TG_pct | 7.986[6.828,9.586] | 7.913[6.781,9.476] | 8.323[7.054,10.055] |
| 49 | Tyr | 0.061[0.053,0.071] | 0.061[0.053,0.071] | 0.063[0.054,0.073] |
| 50 | Val | 0.207[0.181,0.236] | 0.206[0.181,0.235] | 0.208[0.183,0.238] |

sTable 6 Cox regression model to assess associations between metabolic biomarkers of air pollution and osteoarthritis

| No. | Metabolite | HR(95% CI) | P |
| --- | --- | --- | --- |
| 1 | Hbutyrate | 0.972(0.962,0.982) | <0.0001 |
| 2 | Acetate | 0.984(0.973,0.995) | 0.006 |
| 3 | Acetoacetate | 1.008(0.998,1.018) | 0.101 |
| 4 | Acetone | 0.980(0.970,0.990) | <0.001 |
| 5 | Ala | 0.997(0.987,1.007) | 0.547 |
| 6 | Albumin | 0.940(0.931,0.950) | <0.0001 |
| 7 | ApoB_by_ApoA1 | 1.024(1.014,1.035) | <0.0001 |
| 8 | HDL_size | 0.905(0.895,0.915) | <0.0001 |
| 9 | M_VLDL_C | 1.002(0.992,1.013) | 0.637 |
| 10 | IDL_C_pct | 0.935(0.925,0.944) | <0.0001 |
| 11 | M_LDL_C_pct | 0.965(0.957,0.974) | <0.0001 |
| 12 | XL_HDL_C_pct | 1.020(1.010,1.030) | <0.001 |
| 13 | XL_VLDL_C_pct | 0.914(0.904,0.924) | <0.0001 |
| 14 | M_VLDL_CE | 0.981(0.971,0.991) | <0.001 |
| 15 | S_VLDL_CE | 1.049(1.038,1.059) | <0.0001 |
| 16 | L_LDL_CE_pct | 0.997(0.988,1.007) | 0.558 |
| 17 | M_LDL_CE_pct | 1.064(1.053,1.075) | <0.0001 |
| 18 | XL_VLDL_CE_pct | 0.916(0.906,0.926) | <0.0001 |
| 19 | Citrate | 0.942(0.933,0.952) | <0.0001 |
| 20 | Creatinine | 1.033(1.023,1.042) | <0.0001 |
| 21 | Unsaturation | 0.912(0.903,0.922) | <0.0001 |
| 22 | DHA | 0.970(0.960,0.980) | <0.0001 |
| 23 | IDL_FC | 0.978(0.968,0.989) | <0.0001 |
| 24 | M_VLDL_FC | 1.029(1.019,1.040) | <0.0001 |
| 25 | IDL_FC_pct | 0.941(0.932,0.950) | <0.0001 |
| 26 | M_HDL_FC_pct | 0.932(0.922,0.942) | <0.0001 |
| 27 | XL_HDL_FC_pct | 1.059(1.049,1.070) | <0.0001 |
| 28 | Glucose | 1.006(0.996,1.016) | 0.218 |
| 29 | Gly | 0.957(0.947,0.967) | <0.0001 |
| 30 | GlycA | 1.109(1.099,1.120) | <0.0001 |
| 31 | His | 0.988(0.978,0.998) | 0.016 |
| 32 | Ile | 1.034(1.024,1.044) | <0.0001 |
| 33 | Lactate | 0.986(0.976,0.996) | 0.005 |
| 34 | LA_pct | 0.894(0.884,0.903) | <0.0001 |
| 35 | MUFA | 1.096(1.086,1.106) | <0.0001 |
| 36 | Omega_3 | 1.006(0.996,1.016) | 0.207 |
| 37 | Omega_3_pct | 0.968(0.958,0.978) | <0.0001 |
| 38 | Omega_6_by_Omega_3 | 0.997(0.986,1.007) | 0.516 |
| 39 | S_HDL_PL_pct | 1.030(1.020,1.041) | <0.0001 |
| 40 | S_LDL_PL_pct | 0.943(0.934,0.953) | <0.0001 |
| 41 | Pyruvate | 0.998(0.988,1.008) | 0.698 |
| 42 | SFA_pct | 1.079(1.069,1.090) | <0.0001 |
| 43 | Total_P | 1.006(0.996,1.017) | 0.253 |
| 44 | Total_FC | 1.005(0.995,1.016) | 0.338 |
| 45 | LDL_TG | 1.084(1.074,1.095) | <0.0001 |
| 46 | L_HDL_TG | 1.024(1.014,1.034) | <0.0001 |
| 47 | L_LDL_TG | 1.084(1.073,1.094) | <0.0001 |
| 48 | IDL_TG_pct | 1.085(1.074,1.096) | <0.0001 |
| 49 | Tyr | 1.038(1.029,1.048) | <0.0001 |
| 50 | Val | 1.047(1.037,1.057) | <0.0001 |

Models were adjusted for age, sex, race, education, income, physical activity, smoke, alcohol, DASH, BMI, history of diabetes mellitus, hypertension, CVD, and cancer. BMI: Body mass index; DASH: Dietary approaches to stop hypertension; PRS: Polygenic risk score; CVD: Cardiovascular disease.

sTable 7 Mediation of metabolic signature on the association of air pollution with OA

| Exposure | HR(95%CI) | *P* | *E-value* |
| --- | --- | --- | --- |
| Air pollution score |  |  |  |
| Total effect | 1.043(1.021,1.063) | <0.001 | 1.25 |
| Direct effect | 1.034(1.012,1.053) | <0.001 | 1.22 |
| Indirect effect | 1.009(1.008,1.009) | <0.001 | 1.10 |
| Mediation proportion (%) | 21.04(16.52,41.95) | <0.001 |  |
| PM25 |  |  |  |
| Total effect | 1.041(1.018,1.058) | <0.001 | 1.25 |
| Direct effect | 1.032(1.009,1.048) | <0.001 | 1.21 |
| Indirect effect | 1.009(1.009,1.009) | <0.001 | 1.10 |
| Mediation proportion (%) | 21.52(16.73,48.43) | <0.001 |  |
| PM10 |  |  |  |
| Total effect | 1.017(0.991,1.018) | 0.8 | 1.15 |
| Direct effect | 1.012(0.986,1.013) | 0.4 | 1.12 |
| Indirect effect | 1.005(1.004,1.005) | <0.001 | 1.08 |
| Mediation proportion (%) | 29.37(-171.66,940.22) | 0.8 |  |
| PM25-10 |  |  |  |
| Total effect | 1.034(1.026,1.039) | <0.001 | 1.22 |
| Direct effect | 1.031(1.024,1.036) | <0.001 | 1.21 |
| Indirect effect | 1.003(1.002,1.004) | <0.001 | 1.06 |
| Mediation proportion (%) | 8.41(5.78,10.83) | <0.001 |  |
| NO2 |  |  |  |
| Total effect | 1.045(1.021,1.048) | <0.001 | 1.26 |
| Direct effect | 1.037(1.013,1.039) | <0.001 | 1.23 |
| Indirect effect | 1.008(1.008,1.009) | <0.001 | 1.10 |
| Mediation proportion (%) | 18.97(18.91,39.07) | <0.001 |  |
| NOx |  |  |  |
| Total effect | 1.053(1.026,1.064) | <0.001 | 1.29 |
| Direct effect | 1.044(1.018,1.054) | <0.001 | 1.26 |
| Indirect effect | 1.008(1.008,1.009) | <0.001 | 1.10 |
| Mediation proportion (%) | 16.63(15.28,32.26) | <0.001 |  |

sTable 8 Mediation proportion of air pollution metabolic biomarkers on the association of air pollution with osteoarthritis.

| No. | Metabolite | Mediation proportion (%) | P |
| --- | --- | --- | --- |
| 1 | Hbutyrate | -0.66(-4.89,-0.74) | <0.001 |
| 2 | Acetate | -0.58(-7.36,-0.62) | <0.001 |
| 3 | Acetoacetate | 0.56(-0.26,2.88) | 0.667 |
| 4 | Acetone | -0.01(-2.5,-0.08) | <0.001 |
| 5 | Ala | 0.11(-0.55,0.65) | 0.667 |
| 6 | Albumin | 7.43(7.57,34.94) | <0.001 |
| 7 | ApoB_by_ApoA1 | 0.22(0.6,1.34) | <0.001 |
| 8 | HDL_size | 10.12(10.94,56.85) | <0.001 |
| 9 | M_VLDL_C | -0.2(-1.2,0.1) | 0.667 |
| 10 | IDL_C_pct | 5.12(5.17,24.81) | <0.001 |
| 11 | M_LDL_C_pct | 2.84(2.37,11.99) | <0.001 |
| 12 | XL_HDL_C_pct | 1.03(1.53,7.55) | <0.001 |
| 13 | XL_VLDL_C_pct | 2.97(2.1,18.16) | <0.001 |
| 14 | M_VLDL_CE | 1.49(1.16,7.99) | <0.001 |
| 15 | S_VLDL_CE | -1.73(-7.71,-0.75) | <0.001 |
| 16 | L_LDL_CE_pct | 0.15(0.06,0.86) | <0.001 |
| 17 | M_LDL_CE_pct | -1.51(-6.37,-0.51) | <0.001 |
| 18 | XL_VLDL_CE_pct | 2.65(2.02,16.36) | <0.001 |
| 19 | Citrate | 2.72(2.25,9.26) | <0.001 |
| 20 | Creatinine | -1.16(-5.84,-0.92) | <0.001 |
| 21 | Unsaturation | 9.09(7.18,46.95) | <0.001 |
| 22 | DHA | 4.53(3.24,27.31) | <0.001 |
| 23 | IDL_FC | 2.32(2.4,14.12) | <0.001 |
| 24 | M_VLDL_FC | -1.82(-7.25,-1.05) | <0.001 |
| 25 | IDL_FC_pct | 2.07(1.65,11.14) | <0.001 |
| 26 | M_HDL_FC_pct | 9.34(9.59,51.38) | <0.001 |
| 27 | XL_HDL_FC_pct | 4.82(5.91,23.7) | <0.001 |
| 28 | Glucose | 0.18(0.18,1.06) | <0.001 |
| 29 | Gly | 1.09(0.98,5.63) | <0.001 |
| 30 | GlycA | 6.28(4.85,32.52) | <0.001 |
| 31 | His | 0.3(-0.03,2.04) | 0.667 |
| 32 | Ile | -0.31(-0.5,0.72) | 0.667 |
| 33 | Lactate | 1.18(1.42,6.84) | <0.001 |
| 34 | LA_pct | 2.75(1.93,16.64) | <0.001 |
| 35 | MUFA | 0.93(-0.31,8.25) | 0.667 |
| 36 | Omega_3 | -0.92(-1.84,0.39) | 0.667 |
| 37 | Omega_3_pct | 4.1(2.39,23.51) | <0.001 |
| 38 | Omega_6_by_Omega_3 | -0.53(-0.91,2.46) | 0.667 |
| 39 | S_HDL_PL_pct | -1.28(-3.71,-1.95) | <0.001 |
| 40 | S_LDL_PL_pct | -0.36(-3.2,0.43) | 0.667 |
| 41 | Pyruvate | -0.11(-2.73,-0.22) | <0.001 |
| 42 | SFA_pct | -4.18(-20.39,-3.75) | <0.001 |
| 43 | Total_P | -0.72(-0.95,0.03) | 0.667 |
| 44 | Total_FC | -0.6(-2,0.05) | 0.667 |
| 45 | LDL_TG | -1.56(-7.49,-1.51) | <0.001 |
| 46 | L_HDL_TG | -2.14(-7.12,-2.05) | <0.001 |
| 47 | L_LDL_TG | -2.07(-10.23,-1.89) | <0.001 |
| 48 | IDL_TG_pct | 5.98(5.28,28.83) | <0.001 |
| 49 | Tyr | -0.95(-3,0.08) | 0.667 |
| 50 | Val | -1.84(-9.68,-1.68) | <0.001 |

Models were adjusted for age, sex, race, physical activity, smoke, alcohol, DASH, BMI, history of diabetes mellitus, hypertension, CVD, and cancer. BMI: Body mass index; DASH: Dietary approaches to stop hypertension; CVD: Cardiovascular disease.

sTable 9 Sensitive analysis of the associations of air pollution and the related metabolic profiles with osteoarthritis after exclusion of participants with missing covariates.

| Exposure | Model 1 | | Model 2 | | Model 3 | |
| --- | --- | --- | --- | --- | --- | --- |
|  | HR(95%CI) | *P* | HR(95%CI) | *P* | HR(95%CI) | *P* |
| Metabolic profiles of air pollution | | | | | | |
| Each IQR increment | 1.126(1.108,1.144) | <0.0001 | 1.119(1.101,1.138) | <0.0001 | 1.091(1.073,1.109) | <0.0001 |
| Q1 | ref |  | ref |  | ref |  |
| Q2 | 1.082(1.044,1.122) | <0.0001 | 1.078(1.040,1.117) | <0.0001 | 1.064(1.026,1.103) | <0.001 |
| Q3 | 1.153(1.112,1.195) | <0.0001 | 1.145(1.105,1.187) | <0.0001 | 1.114(1.074,1.155) | <0.0001 |
| Q4 | 1.281(1.236,1.328) | <0.0001 | 1.265(1.220,1.312) | <0.0001 | 1.198(1.154,1.243) | <0.0001 |
| P for trend |  | <0.0001 |  | <0.0001 |  | <0.0001 |
| Air pollution score |  |  |  |  |  |  |
| Each IQR increment | 1.027(1.012,1.043) | <0.001 | 1.021(1.006,1.037) | 0.007 | 1.017(1.002,1.033) | 0.028 |
| Q1 | ref |  | ref |  | ref |  |
| Q2 | 1.006(0.971,1.042) | 0.735 | 1.005(0.970,1.041) | 0.803 | 1.000(0.965,1.036) | 0.988 |
| Q3 | 1.052(1.015,1.090) | 0.005 | 1.049(1.012,1.087) | 0.009 | 1.041(1.004,1.078) | 0.028 |
| Q4 | 1.065(1.027,1.104) | <0.001 | 1.052(1.014,1.091) | 0.007 | 1.043(1.006,1.082) | 0.023 |
| P for trend |  | <0.0001 |  | <0.001 |  | 0.005 |

Model 1 was adjusted for age, sex, race, education, and income;

Model 2 was adjusted for Model 1 + physical activity, smoke, alcohol, DASH, and BMI;

Model 3 was adjusted for Model 2 + history of diabetes mellitus, hypertension, CVD, and cancer.

BMI: Body mass index; DASH: Dietary approaches to stop hypertension; CVD: Cardiovascular disease.

sTable 10 Sensitive analysis of the mediation of metabolic signature on the association of air pollution with OA after exclusion of participants with missing covariates

| Exposure | HR(95%CI) | *P* |
| --- | --- | --- |
| Air pollution score |  |  |
| Total effect | 1.027(0.997,1.047) | <0.001 |
| Direct effect | 1.019(1.001,1.039) | <0.001 |
| Indirect effect | 1.008(1.007,1.008) | <0.001 |
| Mediation proportion (%) | 29.14(18.97,76.83) | <0.001 |
| PM25 |  |  |
| Total effect | 1.024(0.994,1.047) | <0.001 |
| Direct effect | 1.017(1.006,1.039) | <0.001 |
| Indirect effect | 1.007(1.007,1.008) | <0.001 |
| Mediation proportion (%) | 30.93(14.72,61.8) | <0.001 |
| PM10 |  |  |
| Total effect | 1.009(0.979,1.034) | 0.4 |
| Direct effect | 1.004(0.974,1.03) | 0.8 |
| Indirect effect | 1.004(1.004,1.005) | <0.001 |
| Mediation proportion (%) | 51.92(-17.41,97.86) | 0.4 |
| PM25-10 |  |  |
| Total effect | 1.031(1.028,1.048) | <0.001 |
| Direct effect | 1.028(1.025,1.045) | <0.001 |
| Indirect effect | 1.003(1.002,1.003) | <0.001 |
| Mediation proportion (%) | 8.62(5.19,10.87) | <0.001 |
| NO2 |  |  |
| Total effect | 1.037(1.015,1.05) | <0.001 |
| Direct effect | 1.03(1.008,1.043) | <0.001 |
| Indirect effect | 1.007(1.007,1.008) | <0.001 |
| Mediation proportion (%) | 20.03(13.83,48.91) | <0.001 |
| NOx |  |  |
| Total effect | 1.041(1.023,1.072) | <0.001 |
| Direct effect | 1.033(1.015,1.064) | <0.001 |
| Indirect effect | 1.007(1.007,1.008) | <0.001 |
| Mediation proportion (%) | 18.77(10.34,33.87) | <0.001 |

sTable 11 Sensitive analysis of the associations of air pollution and the related metabolic profiles with osteoarthritis after exclusion of participants who developed osteoarthritis within 2 years of follow-up.

| Exposure | Model 1 | | Model 2 | | Model 3 | |
| --- | --- | --- | --- | --- | --- | --- |
|  | HR(95%CI) | *P* | HR(95%CI) | *P* | HR(95%CI) | *P* |
| Metabolic profiles of air pollution | | | | | | |
| Each IQR increment | 1.123(1.110,1.137) | <0.0001 | 1.115(1.101,1.129) | <0.0001 | 1.088(1.074,1.103) | <0.0001 |
| Q1 | ref |  | ref |  | ref |  |
| Q2 | 1.097(1.064,1.130) | <0.0001 | 1.090(1.058,1.123) | <0.0001 | 1.075(1.043,1.108) | <0.0001 |
| Q3 | 1.163(1.129,1.198) | <0.0001 | 1.151(1.117,1.186) | <0.0001 | 1.118(1.085,1.152) | <0.0001 |
| Q4 | 1.307(1.269,1.347) | <0.0001 | 1.284(1.246,1.323) | <0.0001 | 1.216(1.179,1.254) | <0.0001 |
| P for trend |  | <0.0001 |  | <0.0001 |  | <0.0001 |
| Air pollution score |  |  |  |  |  |  |
| Each IQR increment | 1.045(1.032,1.058) | <0.0001 | 1.038(1.025,1.051) | <0.0001 | 1.033(1.020,1.046) | <0.0001 |
| Q1 | ref |  | ref |  | ref |  |
| Q2 | 1.023(0.993,1.053) | 0.137 | 1.019(0.990,1.050) | 0.206 | 1.014(0.985,1.044) | 0.353 |
| Q3 | 1.066(1.035,1.097) | <0.0001 | 1.059(1.029,1.091) | <0.001 | 1.051(1.020,1.082) | <0.001 |
| Q4 | 1.101(1.069,1.134) | <0.0001 | 1.087(1.055,1.120) | <0.0001 | 1.075(1.043,1.108) | <0.0001 |
| P for trend |  | <0.0001 |  | <0.0001 |  | <0.0001 |

Model 1 was adjusted for age, sex, race, education, and income;

Model 2 was adjusted for Model 1 + physical activity, smoke, alcohol, DASH, and BMI;

Model 3 was adjusted for Model 2 + history of diabetes mellitus, hypertension, CVD, and cancer.

BMI: Body mass index; DASH: Dietary approaches to stop hypertension; CVD: Cardiovascular disease.

sTable 12 Sensitive analysis of the mediation of metabolic signature on the association of air pollution with OA after exclusion of participants who developed osteoarthritis within 2 years of follow-up

| Exposure | HR(95%CI) | *P* |
| --- | --- | --- |
| Air pollution score |  |  |
| Total effect | 1.045(1.022,1.052) | <0.001 |
| Direct effect | 1.036(1.013,1.042) | <0.001 |
| Indirect effect | 1.008(1.008,1.009) | <0.001 |
| Mediation proportion (%) | 18.79(18.39,40.88) | <0.001 |
| PM25 |  |  |
| Total effect | 1.046(1.03,1.056) | <0.001 |
| Direct effect | 1.037(1.021,1.047) | <0.001 |
| Indirect effect | 1.008(1.008,1.009) | <0.001 |
| Mediation proportion (%) | 18.23(16.63,29.21) | <0.001 |
| PM10 |  |  |
| Total effect | 1.014(1,1.021) | 0.4 |
| Direct effect | 1.01(0.996,1.016) | 0.4 |
| Indirect effect | 1.005(1.004,1.005) | 0 |
| Mediation proportion (%) | 31.87(-887.82,60.51) | 0.4 |
| PM25-10 |  |  |
| Total effect | 1.035(1.025,1.059) | <0.001 |
| Direct effect | 1.032(1.023,1.056) | <0.001 |
| Indirect effect | 1.003(1.002,1.003) | <0.001 |
| Mediation proportion (%) | 7.85(4.32,11.7) | <0.001 |
| NO2 |  |  |
| Total effect | 1.048(1.037,1.063) | <0.001 |
| Direct effect | 1.04(1.028,1.054) | <0.001 |
| Indirect effect | 1.008(1.008,1.009) | <0.001 |
| Mediation proportion (%) | 16.84(14.36,24.11) | <0.001 |
| NOx |  |  |
| Total effect | 1.056(1.041,1.067) | <0.001 |
| Direct effect | 1.048(1.032,1.058) | <0.001 |
| Indirect effect | 1.008(1.008,1.009) | <0.001 |
| Mediation proportion (%) | 14.58(13.01,21.88) | <0.001 |

sTable 13 Sensitive analysis of the associations of air pollution and the related metabolic profiles with osteoarthritis after exclusion of participants who has the history of chronic disease.

| Exposure | Model 1 | | Model 2 | |
| --- | --- | --- | --- | --- |
|  | HR(95%CI) | *P* | HR(95%CI) | *P* |
| Metabolic profiles of air pollution |  |  |  |  |
| Each IQR increment | 1.102(1.082,1.122) | <0.0001 | 1.093(1.074,1.113) | <0.0001 |
| Q1 | ref |  | ref |  |
| Q2 | 1.107(1.065,1.149) | <0.0001 | 1.100(1.059,1.143) | <0.0001 |
| Q3 | 1.154(1.111,1.200) | <0.0001 | 1.143(1.100,1.188) | <0.0001 |
| Q4 | 1.241(1.192,1.292) | <0.0001 | 1.220(1.172,1.271) | <0.0001 |
| P for trend |  | <0.0001 |  | <0.0001 |
| Air pollution score |  |  |  |  |
| Each IQR increment | 1.021(1.005,1.039) | 0.013 | 1.014(0.997,1.031) | 0.101 |
| Q1 | ref |  | ref |  |
| Q2 | 1.016(0.978,1.056) | 0.409 | 1.013(0.975,1.053) | 0.510 |
| Q3 | 1.022(0.983,1.063) | 0.269 | 1.015(0.976,1.055) | 0.461 |
| Q4 | 1.057(1.016,1.100) | 0.006 | 1.041(1.000,1.083) | 0.049 |
| P for trend |  | 0.007 |  | 0.06 |

Model 1 was adjusted for age, sex, race, education, and income;

Model 2 was adjusted for Model 1 + physical activity, smoke, alcohol, DASH and BMI.

BMI: Body mass index; DASH: Dietary approaches to stop hypertension.

sTable 14 Sensitive analysis of the mediation of metabolic signature on the association of air pollution with OA after exclusion of participants who has the history of chronic disease.

| Exposure | HR(95%CI) | *P* |
| --- | --- | --- |
| Air pollution score |  |  |
| Total effect | 1.039(1.021,1.073) | <0.001 |
| Direct effect | 1.03(1.011,1.063) | <0.001 |
| Indirect effect | 1.009(1.009,1.01) | <0.001 |
| Mediation proportion (%) | 23.87(14.12,48.11) | <0.001 |
| PM25 |  |  |
| Total effect | 1.038(1.024,1.078) | <0.001 |
| Direct effect | 1.029(1.015,1.068) | <0.001 |
| Indirect effect | 1.009(1.008,1.01) | <0.001 |
| Mediation proportion (%) | 23.83(13.23,37.89) | <0.001 |
| PM10 |  |  |
| Total effect | 1.009(0.996,1.037) | 0.8 |
| Direct effect | 1.004(0.991,1.031) | 0.8 |
| Indirect effect | 1.005(1.004,1.006) | <0.001 |
| Mediation proportion (%) | 52.77(-156.96,138.65) | 0.8 |
| PM25-10 |  |  |
| Total effect | 1.033(1.022,1.042) | <0.001 |
| Direct effect | 1.03(1.02,1.039) | <0.001 |
| Indirect effect | 1.003(1.003,1.003) | <0.001 |
| Mediation proportion (%) | 8.86(7.7,11.72) | <0.001 |
| NO2 |  |  |
| Total effect | 1.041(1.032,1.07) | <0.001 |
| Direct effect | 1.033(1.024,1.06) | <0.001 |
| Indirect effect | 1.008(1.008,1.009) | <0.001 |
| Mediation proportion (%) | 21.05(13.7,27.09) | <0.001 |
| NOx |  |  |
| Total effect | 1.049(1.04,1.071) | <0.001 |
| Direct effect | 1.04(1.031,1.061) | <0.001 |
| Indirect effect | 1.009(1.008,1.009) | <0.001 |
| Mediation proportion (%) | 18.36(14.1,22.17) | <0.001 |

sTable 15 Sensitive analysis of the associations of air pollution and the related metabolic profiles with osteoarthritis after further adjustment of sleep duration.

| Exposure | Model 1 | | Model 2 | | Model 3 | |
| --- | --- | --- | --- | --- | --- | --- |
|  | HR(95%CI) | *P* | HR(95%CI) | *P* | HR(95%CI) | *P* |
| Metabolic profiles of air pollution | | | | | | |
| Each IQR increment | 1.131(1.118,1.144) | <0.0001 | 1.120(1.106,1.133) | <0.0001 | 1.093(1.079,1.106) | <0.0001 |
| Q1 | ref |  | ref |  | ref |  |
| Q2 | 1.104(1.073,1.136) | <0.0001 | 1.096(1.065,1.128) | <0.0001 | 1.080(1.050,1.111) | <0.0001 |
| Q3 | 1.176(1.143,1.209) | <0.0001 | 1.160(1.128,1.194) | <0.0001 | 1.126(1.094,1.159) | <0.0001 |
| Q4 | 1.329(1.292,1.367) | <0.0001 | 1.299(1.263,1.337) | <0.0001 | 1.229(1.194,1.265) | <0.0001 |
| P for trend |  | <0.0001 |  | <0.0001 |  | <0.0001 |
| Air pollution score |  |  |  |  |  |  |
| Each IQR increment | 1.042(1.030,1.054) | <0.0001 | 1.032(1.020,1.044) | <0.0001 | 1.027(1.015,1.039) | <0.0001 |
| Q1 | ref |  | ref |  | ref |  |
| Q2 | 1.023(0.995,1.051) | 0.116 | 1.018(0.990,1.046) | 0.220 | 1.013(0.985,1.041) | 0.379 |
| Q3 | 1.067(1.038,1.097) | <0.0001 | 1.056(1.027,1.085) | <0.001 | 1.047(1.018,1.077) | 0.001 |
| Q4 | 1.096(1.065,1.127) | <0.0001 | 1.071(1.041,1.102) | <0.0001 | 1.060(1.030,1.090) | <0.0001 |
| P for trend |  | <0.0001 |  | <0.0001 |  | <0.0001 |

Model 1 was adjusted for age, sex, race, education, and income;

Model 2 was adjusted for Model 1 + physical activity, sleep duration, smoke, alcohol, DASH and BMI.

BMI: Body mass index; DASH: Dietary approaches to stop hypertension.

sTable 16 Sensitive analysis of the mediation of metabolic signature on the association of air pollution with OA after further adjustment of sleep duration.

| Exposure | HR(95%CI) | *P* |
| --- | --- | --- |
| Air pollution score |  |  |
| Total effect | 1.038(1.025,1.057) | <0.001 |
| Direct effect | 1.03(1.018,1.049) | <0.001 |
| Indirect effect | 1.008(1.007,1.009) | <0.001 |
| Mediation proportion (%) | 22.74(13.92,29.92) | <0.001 |
| PM25 |  |  |
| Total effect | 1.038(1.019,1.066) | <0.001 |
| Direct effect | 1.029(1.011,1.058) | <0.001 |
| Indirect effect | 1.008(1.007,1.008) | <0.001 |
| Mediation proportion (%) | 22.68(12.32,41.35) | <0.001 |
| PM10 |  |  |
| Total effect | 1.013(0.992,1.027) | 0.4 |
| Direct effect | 1.008(0.988,1.023) | 0.8 |
| Indirect effect | 1.005(1.004,1.005) | <0.001 |
| Mediation proportion (%) | 37.45(-45.98,93.5) | 0.4 |
| PM25-10 |  |  |
| Total effect | 1.032(1.018,1.044) | <0.001 |
| Direct effect | 1.029(1.015,1.041) | <0.001 |
| Indirect effect | 1.003(1.002,1.003) | <0.001 |
| Mediation proportion (%) | 8.62(5.69,14.22) | <0.001 |
| NO2 |  |  |
| Total effect | 1.042(1.026,1.059) | <0.001 |
| Direct effect | 1.033(1.018,1.052) | <0.001 |
| Indirect effect | 1.008(1.007,1.008) | <0.001 |
| Mediation proportion (%) | 19.94(13.13,28.95) | <0.001 |
| NOx |  |  |
| Total effect | 1.047(1.031,1.071) | <0.001 |
| Direct effect | 1.039(1.022,1.064) | <0.001 |
| Indirect effect | 1.008(1.007,1.009) | <0.001 |
| Mediation proportion (%) | 18.05(10.93,28.37) | <0.001 |
